# Identification of tetrahydrocarbazoles as novel multifactorial drug candidates for treatment of Alzheimer's disease

**DOI:** 10.1038/tp.2014.132

**Published:** 2014-12-16

**Authors:** K Honarnejad, A Daschner, A P Gehring, A Szybinska, A Giese, J Kuznicki, F Bracher, J Herms

**Affiliations:** 1 Department of Translational Brain Research, German Center for Neurodegenerative Diseases (DZNE), Munich, Germany; 2 Center for Neuropathology and Prion Research, Ludwig Maximilian University, Munich, Germany; 3 Graduate School of Systemic Neurosciences, Ludwig Maximilian University, Munich, Germany; 4 Department of Pharmacy, Center for Drug Research, Ludwig Maximilian University, Munich, Germany; 5 Laboratory of Neurodegeneration, International Institute of Molecular and Cell Biology, Warsaw, Poland; 6 Munich Cluster for Systems Neurology (SyNergy), Munich, Germany

## Abstract

Alzheimer's disease (AD) is a progressive neurodegenerative brain disorder and the most frequent cause of dementia. To date, there are only a few approved drugs for AD, which show little or no effect on disease progression. Impaired intracellular calcium homeostasis is believed to occur early in the cascade of events leading to AD. Here, we examined the possibility of normalizing the disrupted calcium homeostasis in the endoplasmic reticulum (ER) store as an innovative approach for AD drug discovery. High-throughput screening of a small-molecule compound library led to the identification of tetrahydrocarbazoles, a novel multifactorial class of compounds that can normalize the impaired ER calcium homeostasis. We found that the tetrahydrocarbazole lead structure, first, dampens the enhanced calcium release from ER in HEK293 cells expressing familial Alzheimer's disease (FAD)-linked presenilin 1 mutations. Second, the lead structure also improves mitochondrial function, measured by increased mitochondrial membrane potential. Third, the same lead structure also attenuates the production of amyloid-beta (Aβ) peptides by decreasing the cleavage of amyloid precursor protein (APP) by β-secretase, without notably affecting α- and γ-secretase cleavage activities. Considering the beneficial effects of tetrahydrocarbazoles addressing three key pathological aspects of AD, these compounds hold promise for the development of potentially effective AD drug candidates.

## Introduction

Alzheimer's disease (AD) is the most common cause of dementia in the elderly.^1^ Currently, there is no effective therapeutic modality for the prevention, halting or reversal of AD.^2^ The two principal neuropathological hallmarks of AD are the accumulation of extracellular plaques of β-amyloid (Aβ) peptides and intracellular neurofibrillary tangles of hyperphosphorylated tau protein in the brain. Aβ and tau are thus the prime drug targets for development of disease-modifying therapy in AD.^1^ Nevertheless, the lack of breakthroughs in effective therapy, along with the consistent failure of drug candidates targeting late-stage Aβ and tau pathologies in clinical trials, have recently led to a major shift towards the search for alternative AD drug targets.^3^ Importantly, dysregulated calcium signaling has a central role in AD pathogenesis,^4, 5^ for example, by triggering both Aβ and tau pathology.^6, 7^ Indeed, calcium imaging of AD patient-derived cells^8^ and of neurons from transgenic AD mouse models^9^ indicate that disturbances in endoplasmic reticulum (ER) calcium homeostasis are early events in AD pathogenesis, most likely preceding the clinical manifestations of the disease.^10^ Practically, every gene that is known to directly cause AD or increase susceptibility to it, somehow also affects calcium homeostasis.^4^ Hence, therapeutic interventions aiming at preventing such early calcium dyshomeostasis have been proposed to present a promising opportunity for disease-modifying therapy of AD.^11^ Indeed, pharmacological normalization of ER calcium homeostasis was shown to lower Aβ burden and restore synaptic and cognitive functions in a number of AD mouse models.^12, 13, 14^ Furthermore, due to the multifactorial involvement of ER in the pathogenesis of AD, even minimal levels of therapeutic modulation in the ER may yield tremendous therapeutic efficacy.^15^ In light of such indications and the novelty of this approach, we developed and performed a high-throughput screen for small-molecule compounds that can normalize the enhanced agonist-evoked ER calcium release phenotype in HEK293 cells expressing FAD-linked Presenilin-1 (PS1) mutations. Various mechanisms have been proposed to underlie the FAD-PS1-mediated enhancement of the ER calcium release, for example, enhanced inositol 1,4,5-trisphosphate (IP_3_) receptor and ryanodine receptor (RyR) channel activities, altered sarcoendoplasmic reticulum calcium transport ATPase (SERCA) pump function, decreased capacitative calcium entry and loss of ER passive calcium leakage.^16^ Aside from the controversies in the field as to which of these are the primary causative and which the secondary phenomena, we performed a large-scale phenotypic compound screening. This resulted in the identification of a novel class of chemical structures that normalize the exaggerated calcium release from ER in cells expressing a FAD-PS1 mutation. Stabilization of calcium signaling by the identified lead structure was accompanied by improved mitochondrial function and decreased Aβ peptide production.

## Materials and methods

### Cell culture and cell lines

Human embryonic kidney 293 (HEK293) cells were cultured in Dulbecco's modified eagle medium supplemented with 10% fetal bovine serum and 1% penicillin/streptomycin while being incubated at 37 °C, 5% CO_2_ and 90% humidity. The stable PS1 lines (generously provided by Dr S Lammich) were carrying PS1 variants that were cloned into pcDNA3.1/Zeo(+) and single cells were selected via Zeocin antibiotic resistance.^17^ The PS1 lines were then stably transfected with Yellow Cameleon 3.6 (YC3.6)/pcDNA3 construct (kindly provided by Dr A Miyawaki) and single cells were respectively isolated by G418 antibiotic resistance leading to the generation of double stable lines. The amyloid precursor protein (APP)-, C99- and APPsw/PS1-M146L-overexpressing HEK293 lines were kindly provided by Dr S. Lichtenthaler and Dr H. Steiner and cultured as previously described.^18, 19^

### Automated high-throughput FRET-based calcium imaging and image analysis

HEK293 cells stably expressing PS1-M146L and YC3.6,^20^ were seeded at 13 000 cells per well in 40 μl of growth medium on collagen-coated 384-well CellCarrier plates (Perkin Elmer, Rodgau, Germany). After 6 h, using an automated pipetting robot (Bravo, Agilent Technologies, Santa Clara, CA, USA), library compounds were added to each well at the final concentration of 10 μM in 1% dimethyl sulfoxide (DMSO), each in four replicates. All plates contained Thapsigargin (TP; 1 μM; Calbiochem, Darmstadt, Germany), cyclopiazonic acid (CPA; 20 μM; Calbiochem), 3,4,5-trimethoxybenzoic acid 8-(diethylamino)octyl ester (TMB-8; 50 μM; Sigma-Aldrich, Taufkirchen, Germany) and Bepridil (20 μM; Sigma-Aldrich) as positive controls reducing the amount of calcium release from ER, as well as untreated and DMSO vehicle-treated wells. After 24 h using the pipetting robot, DRAQ5 (Biostatus, Leicestershire, UK), a far-red fluorescent nuclear dye, was added to each well at the final concentration of 500 nM. After 2 h, plates were measured for carbachol (CCh)-induced calcium release using Opera high-throughput confocal imaging platform (Perkin Elmer Cellular Technologies GmbH, Hamburg, Germany). Throughout imaging of the entire plate, 37 °C temperature, 5% CO_2_ and 90% humidity was maintained in the plate chamber. Using a 442 nm laser, YC3.6 was excited and its CFP and YFP emissions were separated, respectively, using 483/35 nm and 540/75 nm filters. In addition, using a 640 nm laser, DRAQ5 dye was excited and its emission was collected by 690/50 nm filter to locate the nuclei. Imaging was performed with a × 20 water immersion autofocus objective. The duration of the entire time-lapse calcium imaging for each well was 23.5 s. This was achieved by imaging at 2.5 s interval resolution before dispensing CCh (for 5 s) to monitor the basal calcium levels. Next, the CCh-induced calcium rise and decay were monitored for 18.5 s post dispensing. Imaging was performed first at 1 s interval resolution immediately after dispensing (for 5 s) and subsequently at 2.5 s interval resolution (for 12.5 s). During dispensing, 10 μl of CCh (Calbiochem) diluted in HBSS (10 μM) was injected to each well concurrent with calcium imaging by an automated dispensing unit which is part of the Opera platform. Imaging was performed sequentially for all the 384 wells. Using Acapella software (Perkin Elmer Cellular Technologies GmbH), an automated image analysis tool was developed to convert fluorescent signals to numerical values. Here, DRAQ5 and YC3.6 signals were used, respectively, to detect single-cell nuclei and single-cell boundaries over the entire course of time-lapse calcium imaging. After assigning each cell to its segmented nuclei and excluding the cells positioned at the edges of the imaging frames, calcium transients for every cell were monitored by plotting the kinetics of YFP/CFP versus time and normalizing the signals using the equation, Δ*F*/*F*_0_=(*F*−*F*_0_)/*F*_0_, where *F* is the measured fluorescence signal at any given time and *F*_0_ is the average fluorescence signal of the time points preceding CCh application. The peak amplitude of calcium rise upon CCh injection was the output of automated image analysis at single-cell level. Nonresponsive cells to CCh were excluded from analysis by setting an arbitrarily defined threshold. The average peak amplitude of all responsive cells in each well was calculated as the final readout in this assay.

### Mitochondrial membrane potential TMRM assay

The measurement method for mitochondrial membrane potential with tetramethylrhodamine methyl ester (TMRM) dye was adapted from Scaduto *et al.*^21^ HEK293 cells were seeded at the density of 50 000 cells per well on collagen/poly-L-lysine (PLL)-coated 96-well plates (Advanced-TC plates, Greiner Bio-One GmbH, Frickenhausen, Germany) and incubated for 24 h. Next, the cells were loaded with 50 nM TMRM (Invitrogen, Carlsbad, CA, USA) dye in the presence of either tetrahydrocarbazoles (10 μM), positive control Dimebon (10 μM; Sigma-Aldrich) or DMSO vehicle, which were pre-incubated on the cells 1 h before the addition of TMRM dye. After 30 min, each well was washed three times using phosphate-buffered saline. Fresh medium containing each of the corresponding tested compounds (10 μM) was added into the wells. Live cell image acquisition was performed using inverted confocal microscope LSM510 with × 25 magnification (Carl Zeiss MicroImaging GmbH, Jena, Germany) and the images were analyzed using ImageJ software (NIH, Bethesda, MD, USA) to quantify the intensity of TMRM fluorescence signal. All the measurements were performed with at least eight replicates.

### Aβ measurements

The levels of three different Aβ species (Aβ38, Aβ40 and Aβ42) were measured using sandwich enzyme-linked immunosorbent assay (ELISA). Pools of HEK293 cells stably transfected with either APPsw/PS1-M146L or APP were used to study the effect of compounds on Aβ generation. According to Page *et al.*,^19^ cells were seeded at a density of 200 000 cells per well in collagen/poly-L-lysine (PLL)-coated 24-well plates and incubated for 24 h in the growth medium. Next, the medium was exchanged with 500 μl of fresh medium containing either the tested compounds, or the positive controls DAPT (10 μM, Calbiochem), Sulindac sulfide (50 μM, Sigma-Aldrich), Bepridil (30 μM, Sigma-Aldrich)^18^ or DMSO vehicle. After 16 h conditioned medium was collected and the levels of secreted Aβ38, Aβ40 and Aβ42 fragments were quantified using ‘Human (6E10) Abeta 3-Plex' sandwich ELISA immunoassay kit (Meso Scale Discovery, Rockville, MD, USA) according to the instructions of the manufacturer. In brief, 150 μl of blocker reagent was added to each well and incubated for 1 h at room temperature, followed by 3 × washing using TRIS wash buffer. Next, 25 μl of detection antibody was added to each well. At appropriate dilution, each of the samples or calibration standards were added to separate wells of ELISA plate and incubated for 2 h at room temperature, followed by 3 × washing using TRIS wash buffer. Finally, 150 μl of read buffer was added to the wells. The light emission after electrochemical stimulation was measured using Sector Imager 2400 reader (Meso Scale Discovery). On the basis of the values generated with calibration standards, corresponding concentrations of Aβ species were calculated using the Meso Scale Discovery Workbench software. All the measurements were performed with at least two replicates.

### sAPPα and sAPPβ measurements

Levels of sAPPα and sAPPβ fragments were measured using sandwich ELISA adapted from Colombo *et al.*^22^ Wild-type HEK293 cells were seeded at a density of 200 000 cells per well in collagen/poly-L-lysine (PLL)-coated 24-well plates and incubated for 24 h in the growth medium. Next, the medium was exchanged with 500 μl of fresh medium containing either compounds or vehicle. After 16 h, conditioned medium was collected and the levels of secreted sAPPα and sAPPβ fragments were quantified using sAPPα/sAPPβ sandwich ELISA immunoassay kit (Meso Scale Discovery) according to the instructions of the manufacturer. Briefly, 150 μl of blocker reagent was added to each well of the ELISA plate and incubated for 1 h at room temperature, followed by 3 × washing using TRIS wash buffer. Next, 25 μl of samples or calibration standards were added to separate wells of ELISA plate and incubated for 1 h at room temperature, followed by 3 × washing using TRIS wash buffer. Then, 25 μl of detection antibody was added to each well and incubated for 1 h at room temperature, followed by 3 × washing using TRIS wash buffer. Finally, 150 μl of read buffer was added to the wells. The light emission after electrochemical stimulation was measured using Sector Imager 2400 reader (Meso Scale Discovery). On the basis of the values generated with calibration standards, corresponding concentrations of sAPPα and sAPPβ were calculated using the Meso Scale Discovery Workbench software. All the measurements were performed in four replicates.

### Statistical data analysis

GraphPad Prism 5.0 b (GraphPad Software, San Diego, CA, USA) was used for statistical analysis of the data. For comparison and *P*-value determination, we used one-way analysis of variance (ANOVA) method, followed by Dunnett's multiple comparison test. All the data are represented as means±s.d. Differences were considered statistically significant if *P*<0.05.

## Results

### Discovery of a novel lead structure from a high-throughput compound screen targeting disrupted ER calcium homeostasis

In light of growing evidence towards the role of impaired intracellular store calcium homeostasis in the pathogenesis of Alzheimer's disease,^23^ here, we screened for low-molecular-weight compounds that can normalize the disrupted ER calcium homeostasis. We chose the potentiated agonist-evoked ER calcium release in FAD-PS1-expressing cells as a robust phenotypic model to target ER calcium dyshomeostasis for AD drug discovery.

**Figure 1:**
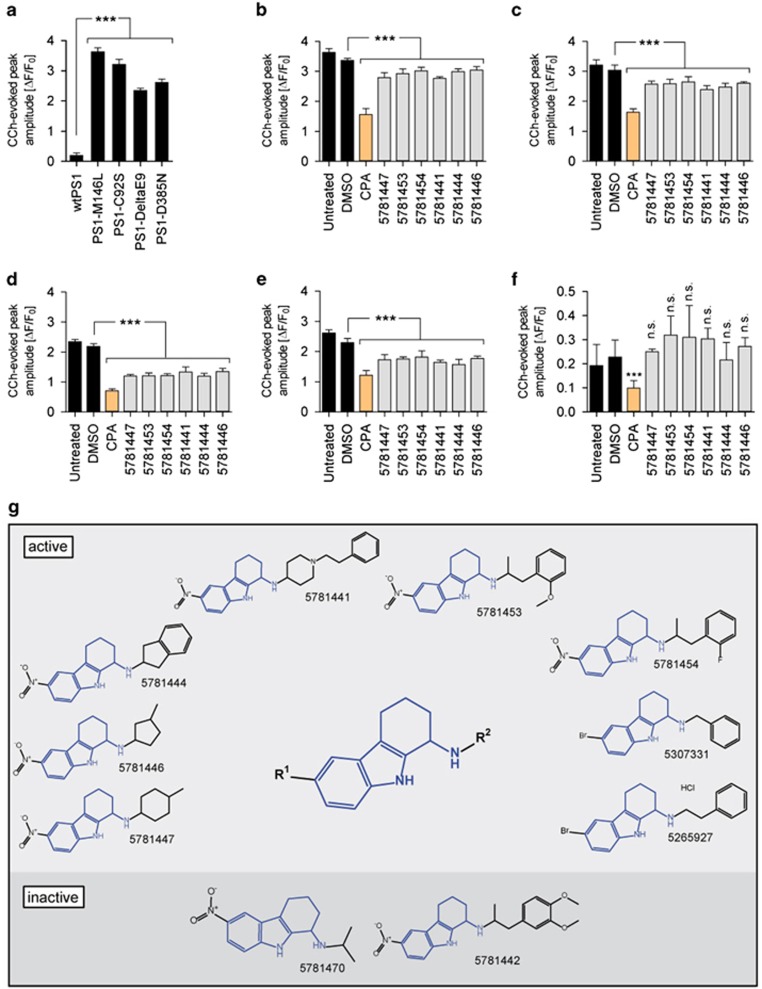
Tetrahydrocarbazole analog screening hits/lead structure and their effects on FAD-PS1-mediated disrupted ER calcium release. (**a**) The peak amplitude of CCh-evoked calcium release in HEK293 cells expressing wild-type PS1, FAD-linked (PS1-M146L, PS1-C92S and PS1-DeltaE9) or a γ-secretase deficient (PS1-D385N) PS1 mutations. The effect of six tetrahydrocarbazole hits identified from the primary screen at 10 μM in (**b**) PS1-M146L, (**c**) PS1-C92S, (**d**) PS1-DeltaE9, (**e**) PS1-D385N and (**f**) wild-type PS1-expressing HEK293 cells on the peak amplitude of CCh-evoked calcium release. CPA, an inhibitor of calcium-dependent ATPases, was used as a positive control. (**g**) The tetrahydrocarbazole lead structure, identified from a high-throughput compound screen for substances normalizing the exaggerated CCh-evoked calcium release in PS1-M146L HEK293 cells. Illustrated are chemical structures of the 10 tetrahydrocarbazole analogs present in the entire screened compound library. The upper and lower panels indicate, respectively, eight active and two inactive analogs of the lead structure. Compounds capable of reducing the peak amplitude of CCh-induced calcium release to <90% of DMSO-treated controls (normalized ER calcium <0.9) were regarded as active hits. (n.s., non-significant; ***P<0.001; *n*=4). CCh, carbachol; CPA, cyclopiazonic acid; DMSO, dimethyl sulfoxide; ER, endoplasmic reticulum; FAD-PS1, familial Alzheimer's disease presenilin 1.

All mutant PS1 lines tested revealed remarkably enhanced calcium release when compared with wild-type PS1-expressing cells (Figure 1a). A phenotypic screening for compounds that are capable of dampening the potentiated CCh-evoked ER calcium release in PS1-M146L HEK293 cells was subsequently performed. Screening a diverse compound library comprising 20 000 small molecules led to the discovery of 1-amino-1,2,3,4-tetrahydrocarbazoles as a novel lead structure. Six recognized representatives of this structural motif in the library which showed activity in the screen (Figure 1b), remained active across several other mutant PS1-expressing lines (Figures 1c–e). Importantly, the amplitude of CCh-evoked calcium release in wild-type PS1-expressing cells was not significantly altered (Figure 1f). For the primary screen, a compound was regarded as active if it reduced the peak amplitude of CCh-induced calcium release to <90% of DMSO-treated controls (normalized ER calcium <0.9).

The discovered lead structure, identified as and hereafter called tetrahydrocarbazoles, consists of a core moiety having two variable R groups, shown as R^1^ and R^2^ (Figure 1g). Comprehensive data mining revealed that the entire compound library contained 10 analogs of the lead structure, 8 of which were found to be active in the screen (Figure 1g).

### Tetrahydrocarbazoles attenuate the FAD-PS1 mediated exaggerated ER calcium release

**Figure 2:**
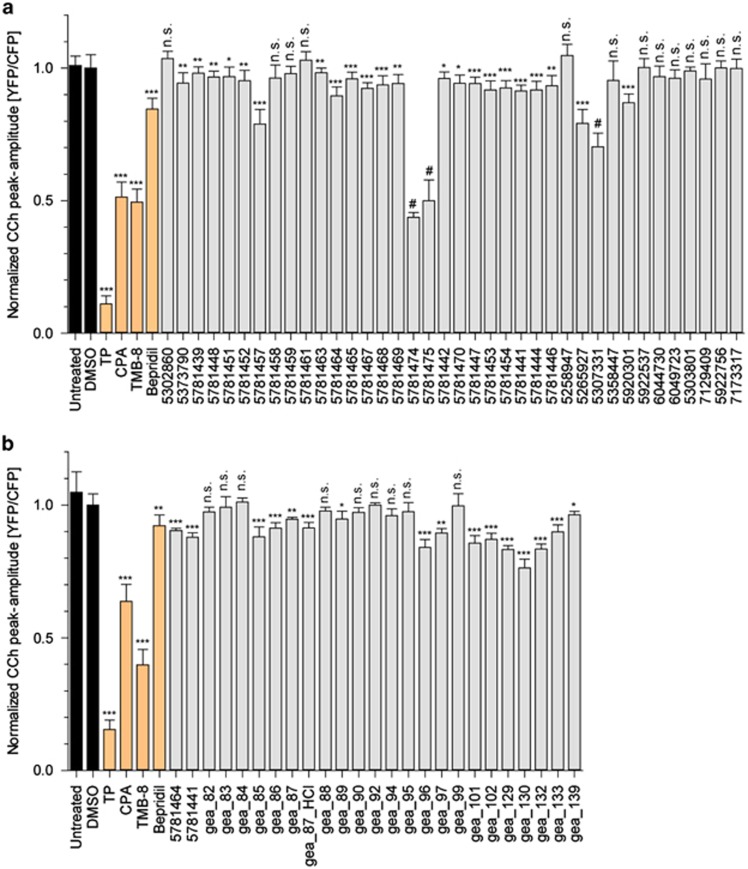
The effects of tetrahydrocarbazoles and analogs on the FAD-PS1-mediated enhanced ER calcium release. (**a**) The activity of commercially available and (**b**) synthesized tetrahydrocarbazoles and analogs tested at 10 μM in PS1-M146L HEK293 cells. The presented values indicate the normalized peak amplitude of CCh-evoked calcium release for cells treated with each compound for 24 h relative to the peak amplitude of DMSO-treated control (normalized ER calcium). Compounds marked with # symbol possess a certain level of toxicity, which interferes with the calcium release measurement in this assay. TP (1 μM), CPA (20 μM), TMB-8 (50 μM) and Bepridil (20 μM), all lowering the amount of calcium release from ER, were used as positive controls. (n.s. non-significant; *P<0.05, **P<0.01 and ***P<0.001; *n*=4). CCh, carbachol; CPA, cyclopiazonic acid; DMSO, dimethyl sulfoxide; ER, endoplasmic reticulum; FAD-PS1, familial Alzheimer's disease presenilin 1; TMB, 3,4,5-trimethoxybenzoic acid 8-(diethylamino)octyl ester; TP, Thapsigargin.

In order to explore the contribution of different R^1^ and R^2^ groups to the activity of the lead structure, we further tested 28 commercially available tetrahydrocarbazole analogs and related structures. We also validated the activity of the 10 structures previously identified from the primary screen (Figure 2a and Supplementary Figure S1). On the basis of the structure–activity relationship knowledge gained, we synthesized 23 further derivative structures with the aim of reaching an improved efficacy (Figure 2b and Supplementary Figure S2; for details about the synthesis, see Supporting Information). Replacement of nitro group at R^1^ position with other electron-withdrawing substituents, for example, halogens, trifluoromethyl and cyano groups, maintains the activity of the lead structure, while other small substituents, for example, hydrogen, lead to the loss of activity. Aliphatic residues at R^2^ position (for example, 5781439, 5781448, 5781457, gea_87) diminish that effect, while additional attachment of an aromatic motif (for example, phenyl group) is beneficial to the activity (for example, 5781464, 5781441).

### Tetrahydrocarbazoles increase the mitochondrial membrane potential

**Figure 3:**
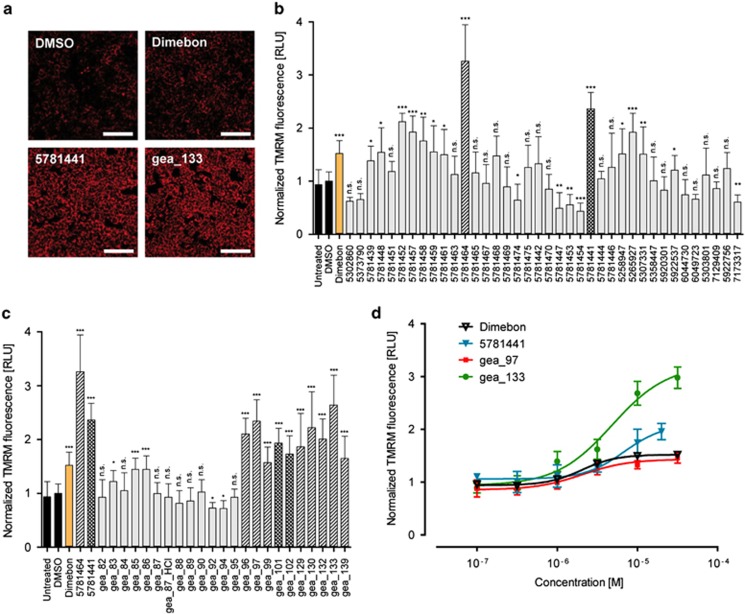
The effect of tetrahydrocarbazole analogs on mitochondrial membrane potential. (**a**) Representative TMRM-staining images of HEK293 cells pretreated for 1 h with the indicated tetrahydrocarbazoles (10 μM) or Dimebon (10 μM) as a positive control, relative to DMSO-treated control (scale bar, 100 μm). (**b**) Quantification of the average TMRM-staining signals showing relative intensity for commercially available analogs of the tetrahydrocarbazole lead structure upon 1 h pretreatment of HEK293 cells (10 μM). The bars highlighted with single- and double-stripes represent the most active compounds 5781464 and 5781441, which, respectively, possess *N*-(1-benzylpiperidin-4-yl) and *N*-(1-phenethylpiperidin-4-yl) groups at their R^2^ position. (**c**) Quantification of average TMRM intensity for synthesized tetrahydrocarbazole derivatives tested at 10 μM. The marked single-striped bars represent the analogous structures similar to 5781464, possessing *N*-(1-benzylpiperidin-4-yl) at their R^2^ position, and double-striped bars represent derivative compounds similar to 5781441, which contain *N*-(1-phenethylpiperidin-4-yl) group at the R^2^ position. (**d**) Quantification of average dose-dependent TMRM relative intensities for three select tetrahydrocarbazoles tested at six different concentrations relative to Dimebon. The EC_50_ of all analogs tested lies at low micromolar range. All the values are normalized to DMSO value, which is set to 1. (n.s., non-significant; *P<0.05, **P<0.01 and ***P<0.001; *n*=8). DMSO, dimethyl sulfoxide; TMRM, tetramethylrhodamine methyl ester.

It has been demonstrated that ER and mitochondria are physically and functionally interdependent.^24^ Constitutive calcium release from IP_3_R to mitochondria is a crucial mechanism involved in mitochondrial function.^25^ Indications suggest that FAD-PS mutations affect the physical interaction between ER and mitochondria,^26^ leading to altered shuttling of calcium between the two organelles and modulating the mitochondrial calcium uptake.^27^ Thus, in the next set of experiments, we explored whether the modulation of ER calcium homeostasis by the lead structure also affects mitochondrial function. To that end, we analyzed mitochondrial membrane potential as an important parameter for addressing mitochondrial activity. We used TMRM dye, a fluorescent rhodamine derivative, to monitor mitochondrial membrane potential.^21^ Indeed, pretreatment of HEK293 cells for 1 h with several tetrahydrocarbazoles led to a remarkable increase in the mitochondrial membrane potential, measured by the TMRM fluorescence signal (Figures 3a and b). At 10 μM, the increases in mitochondrial membrane potential after treatment with many of the analogs were comparable or even superior to that for Dimebon, a known enhancer of mitochondrial activity^28^ (Figures 3a and b). We particularly found that compounds 5781464 and 5781441, respectively, possessing *N*-(1-benzylpiperidin-4-yl) and *N*-(1-phenethylpiperidin-4-yl) groups at their R^2^ position, were among the most active compounds both in terms of efficacy and potency (Figures 3b and d). Therefore, in several lead structure derivatives that we synthesized, the R^2^ position remained incorporating *N*-(1-benzylpiperidin-4-yl) or *N*-(1-phenethylpiperidin-4-yl) groups, whereas we varied the groups at the R^1^ position to explore their influence on the activity of the lead structure (Supplementary Figure S2). Indeed the latter analogs were also among the most active synthesized compounds in enhancing mitochondrial function (Figure 3c). Therefore, we concluded that the highest activity in terms of improving mitochondrial membrane potential is achieved if the lead structure possesses *N*-(1-benzylpiperidin-4-yl) or *N*-(1-phenethylpiperidin-4-yl) groups at the R^2^ position, given that the R^1^ position incorporates electron-withdrawing residues. Exemplarily, the EC_50_ for one of the most promising synthesized derivatives of the lead structure (gea_133; R^1^: cyano) was determined to be at the therapeutically relevant value of 4.84 μM (Figure 3d). Moreover, the efficacy of compound gea_133 was remarkably higher than Dimebon, especially at concentrations beyond 1 μM (Figure 3d).

### Tetrahydrocarbazoles lower Aβ peptide production

**Figure 4:**
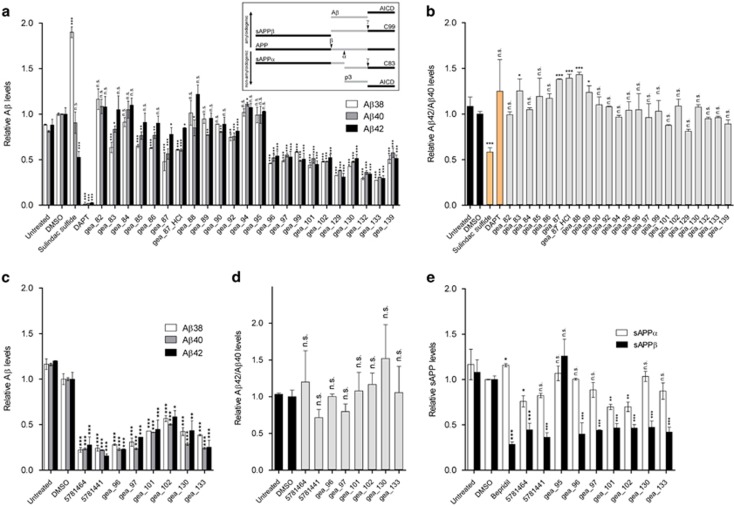
The effect of tetrahydrocarbazoles on APP processing. (**a**) Relative Aβ38, Aβ40 and Aβ42 levels, decreased after 16 h treatment with synthesized tetrahydrocarbazoles at 10 μM in HEK293 cells coexpressing APPsw and PS1-M146L. Sulindac sulfide (50 μM), a γ-secretase modulator, and DAPT (10 μM), a γ-secretase inhibitor, were used as positive controls. Inside the box, a schematic illustration of APP processing by α-, β- and γ-secretase is presented. (**b**) Relative Aβ42/Aβ40 ratios calculated from **a**. Treatment with the majority of tetrahydrocarbazoles does not alter Aβ42/Aβ40 ratio, whereas positive control Sulindac sulfide significantly lowers Aβ42/Aβ40 ratio. (**c**) Relative Aβ38, Aβ40 and Aβ42 levels are decreased after 16 h treatment with select tetrahydrocarbazoles at 10 μM in HEK293 cells overexpressing wild-type APP. (**d**) Relative Aβ42/Aβ40 ratios calculated from **c**. Treatment with select tetrahydrocarbazoles tested does not alter Aβ42/Aβ40 ratio. (**e**) Relative sAPPα and sAPPβ levels after 16 h compound treatment in wild-type HEK293 cells. Treatment with select tetrahydrocarbazole derivatives does not (or only marginally) affect secreted sAPPα levels, whereas secreted sAPPβ fragment levels are remarkably decreased. All the values are normalized to the value of DMSO, which is set to 1. (n.s., non-significant; *P<0.05, **P<0.01 and ***P<0.001; *n*=2). APP, amyloid precursor protein; DMSO, dimethyl sulfoxide; PS1, presenilin 1.

**Figure 5:**
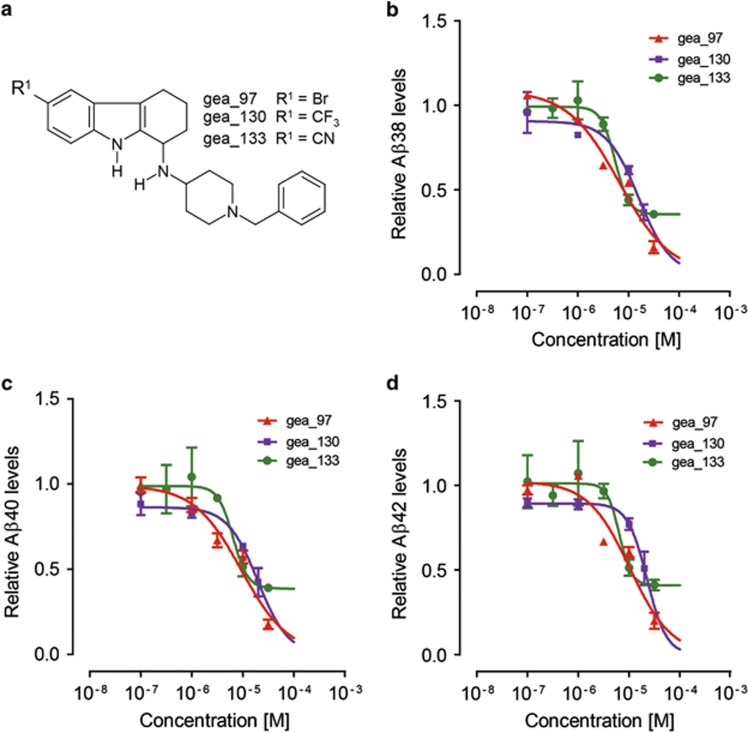
Dose-dependent effects of tetrahydrocarbazole derivatives on Aβ production. (**a**) Chemical structure of three promising synthesized tetrahydrocarbazole analogs. The effect of the three select synthesized tetrahydrocarbazoles on the production of (**b**) Aβ38, (**c**) Aβ40 and (**d**) Aβ42 peptides tested at six different concentrations in APPsw/PS1-M146L-expressing HEK293 cells. The IC_50_ of all Aβ species for all derivative structures tested lies at low micromolar range. All the values are normalized to the value of DMSO, which is set to 1. DMSO, dimethyl sulfoxide.

**Figure 6:**
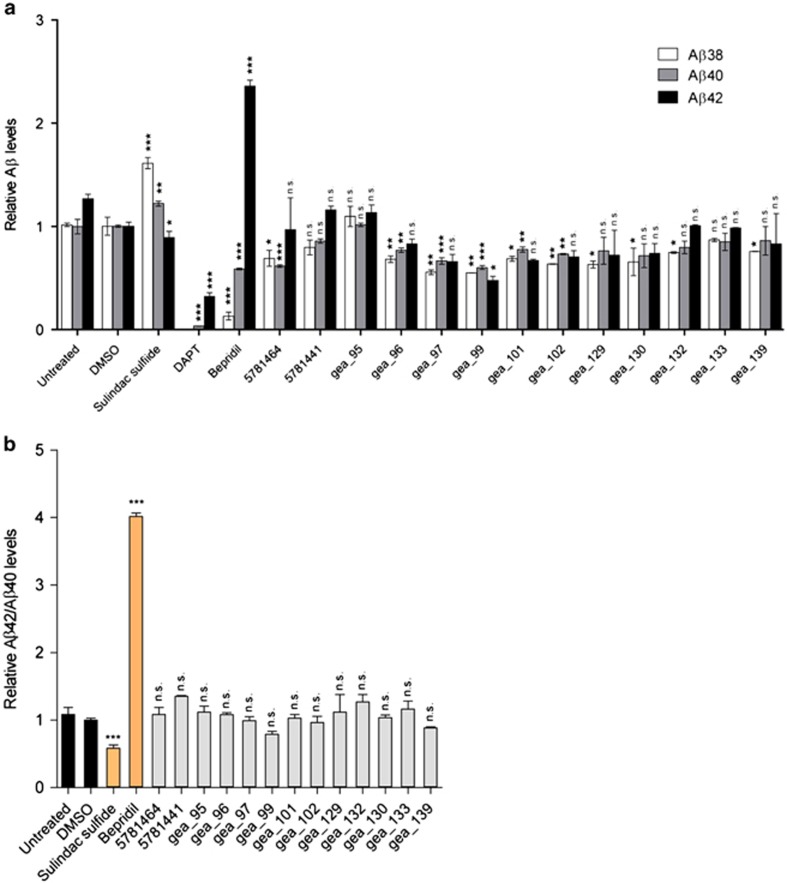
The effect of synthesized tetrahydrocarbazoles and analogs on γ-secretase cleavage activity in HEK293-C99 cells. (**a**) Relative Aβ38, Aβ40 and Aβ42 levels after 16 h treatment with select tetrahydrocarbazole derivatives at 10 μM in HEK293-C99 cells. Sulindac sulfide (50 μM), Bepridil (30 μM) and DAPT (10 μM), respectively, a γ-secretase modulator, an iGSM and a γ-secretase inhibitor, were used as positive controls. All the values are normalized to the value of DMSO, which is set to 1. (n.s., non-significant; *P<0.05, **P<0.01 and ***P<0.001; *n*=2). (**b**) Relative Aβ42/Aβ40 ratios calculated from **a**. Treatment with tested analogs does not alter Aβ42/Aβ40 ratios, whereas positive controls Sulindac sulfide and Bepridil, respectively, lead to a significant decrease and increase in Aβ42/Aβ40 ratios. All the values are normalized to the value of DMSO, which is set to 1. (n.s., non-significant; *P<0.05, **P<0.01 and ***P<0.001; *n*=2). DMSO, dimethyl sulfoxide.

FAD-PS mutations are well known to promote the production of Aβ42 peptide.^29^ Therefore, next, we studied the impact of tetrahydrocarbazoles on the production of Aβ peptides. Modulation of intracellular calcium homeostasis directly affects Aβ production.^30^ Thus, we hypothesized that normalizing the disrupted ER calcium homeostasis may additionally result in lowered Aβ production. Indeed, we detected remarkably decreased levels of secreted Aβ38, Aβ40 and Aβ42 peptides upon 16 h treatment of HEK293 cells expressing either APPsw/PS1-M146L or wild-type APP with the lead structure analogs at 10 μM (Figures 4a and c and Supplementary Figure S3). The IC_50_ of the select promising analogs in terms of decreasing levels of all three Aβ species lies in the low micromolar range (Figures 5b–d). However, compound treatment in both the cell lines did not affect the Aβ42/Aβ40 ratio for most analogs, suggesting that the identified lead structure is not a γ-secretase modulator (Figures 4b and d and Supplementary Figure S4). To investigate the γ-cleavage of APP independently from its β-cleavage, we used HEK293 cells expressing C99, the β-cleaved carboxy (C)-terminal fragment of APP and the substrate for γ-secretase. Here, we observed that treatment of HEK293-C99 cells with the majority of the tetrahydrocarbazoles tested, did not (or only marginally) affect the production of Aβ38, Aβ40 and Aβ42 (Figure 6a). Moreover, Aβ42/Aβ40 ratios remained unaffected upon exposure of HEK293-C99 cells with the tetrahydrocarbazoles (Figure 6b). Taken together, these results support the conclusion that the detected decrease in Aβ peptide levels is not a γ-secretase-dependent phenomenon. In accordance, we postulated that reduced β-cleavage of APP may contribute to lowered Aβ generation. Hence, we measured the levels of sAPPα and sAPPβ, the first cleavage products of APP, generated by α-secretase and β-secretase, respectively. Indeed, we detected significantly decreased levels of secreted sAPPβ, while sAPPα levels were mostly unaffected (or only mildly reduced) upon treatment of wild-type HEK293 cells with the tested substances (Figure 4e). These results imply that the attenuated Aβ production caused by the compounds is mediated through decreased cleavage of APP by β-secretase. The structure–activity relationship analysis among the lead structure analogs in terms of lowering Aβ production was comparable to their determined structure–activity relationship for increasing mitochondrial membrane potential. We found that analogs incorporating electron-withdrawing residues at the R^1^ position, in combination with *N*-(1-benzypiperidin-4-yl) or *N*-(1-phenethylpiperidin-4-yl) at R^2^ position show the most robust reduction in Aβ production (Figure 4a).

## Discussion

Dysfunction and loss of neurons and synapses are by far the best available correlates of cognitive deficits in AD patients.^31, 32^ Regulation of calcium homeostasis is essential for neuronal function and synaptic activity.^33^ Early-stage aberrant calcium signaling in AD is proposed to underlie the late-stage synaptic dysfunction and memory deficits.^34^ Notably, alterations in ER calcium channels were found to correlate with neurofibrillary and Aβ pathologies of AD brain.^7^ Moreover, altered calcium homeostasis in the peripheral tissues was proposed as diagnostic biomarkers of mild AD.^35, 36^ Furthermore, the beneficial effects of memantine, an NMDA receptor antagonist, for the treatment of moderate-to-severe AD patients reinforce the relevance of calcium-signaling-targeted AD therapy.^37^ Indeed, pharmacological normalization of disrupted ER calcium homeostasis by blocking hyperactivated RyR channels with dantrolene was demonstrated to be associated with decreased Aβ burden, increased PSD-95 expression and improvements in learning and memory in APPsw-expressing mouse model of AD.^13^ Altogether, given the central role of calcium both in triggering the early disease-initiating pathomechanisms as well as accelerating the AD pathology at later stages,^38^ targeting altered calcium signaling presents an attractive target for both AD prevention and treatment. Accordingly, we developed and performed a high-throughput screen for compounds that can normalize the aberrant ER calcium homeostasis phenotype caused by FAD-linked PS1 mutations. This approach led to the discovery of tetrahydrocarbazoles, a novel lead structure capable of lowering the exaggerated CCh-evoked ER calcium release in FAD-PS1 cells.

In addition to the stabilization of ER calcium homeostasis, we observed that tetrahydrocarbazoles can improve mitochondrial function, measured by increased mitochondrial membrane potential. Mitochondrial dysfunction is proposed to act as a trigger in AD pathogenesis and a contributing factor to both onset and progression of the disease.^39^ In addition to aberrant calcium homeostasis, mitochondrial dysfunction is an additional early event in the course of AD, thus presenting an attractive target for preventative therapy.^40^ FAD-PS1 mutations have been shown to cause abnormal changes in mitochondrial morphology and sensitivity to apoptosis upon mitochondrial failure.^41, 42^ Neuronal cells harboring FAD-PS1 display decreased mitochondrial membrane potential under oxidative stress conditions.^41^ Yet another study reported no significant relative mitochondrial membrane potential differences between fibroblasts derived from FAD-PS1 mutation carriers versus controls.^43^ Growing body of evidence indicates that the ER–mitochondria physical interfaces and calcium shuttling between the two organelles through IP_3_ receptors have a crucial role in the regulation of mitochondrial function,^25^ which appears to be affected in AD.^26, 27^ Treatment with many of the lead structure derivatives resulted in a larger increase in the mitochondrial membrane potential than treatment with Dimebon. The latter suggests higher efficacy for tetrahydrocarbazoles compared with Dimebon.

Extensive literature underlines the role of FAD-PS mutations in altering the proteolytic processing of APP, which, in turn, leads to selective increase in the formation of longer Aβ42 versus the shorter Aβ40 peptides.^29, 44^ We found that treatment with many tetrahydrocarbazole analogs results in notably less Aβ38, Aβ40 and Aβ42 production in two different cell lines. Yet, Aβ42/Aβ40 ratios remained largely unchanged. The latter indicates that the identified lead structure does not possess the properties of a γ-secretase modulator. γ-Secretase modulators are characterized by decreased production of longer Aβ species (for example, Aβ42) accompanied by increased generation of shorter Aβ species (for example, Aβ38 and Aβ40), resulting in the lowering of Aβ42/Aβ40 ratio.^45^ Although there is evidence that γ-secretase activity may be affected by calcium ions,^46^ the detected decreases in Aβ levels were not predominantly caused by γ-secretase inhibition. The evidence for the latter comes from the experiments with HEK293-C99 cells, suitable for exclusively addressing the γ-secretase cleavage of APP (independently from β-secretase activity). In HEK293-C99 cells, we detected unchanged or only slightly decreased Aβ levels upon treatment with the derivative structures tested. These minor reductions in Aβ levels caused by some lead structure derivatives can be due to the fact that calcium ions can modulate the γ-secretase activity to some extent.^46^ However, such minor effects are unlikely to account for the remarkable decrease in Aβ levels observed after the treatment of APPsw/PS1-M146L and APP-expressing cells with tetrahydrocarbazole analogs. Our findings rather suggest that lowered Aβ production is mainly attributed to decreased β-cleavage of APP. We detected remarkably decreased sAPPβ levels upon exposure of HEK293 cells with the lead structure derivatives. Indeed, it has been demonstrated that calcium directly enhances the proteolytic activity of β-secretase (BACE1).^47^ Therefore, it is plausible that the stabilization of ER calcium homeostasis by tetrahydrocarbazoles results in lowered BACE1 activity and consequently decreased Aβ production. The decrease in sAPPβ was not accompanied by an increase in sAPPα, indicating that the lead structure does not alter the α-secretase cleavage activity. The lack of inverse coupling between α- and β-secretase activities in frequently used cell lines, for example, HEK293 cells, may explain our finding that the compounds lower sAPPβ generation without changing sAPPα levels.^22^ The observation that most tetrahydrocarbazoles do not affect Aβ and sAPPα levels, respectively, in HEK293-C99 and wild-type HEK293 cells (for example, gea_133), indicates that the decreased Aβ production through lowering β-cleavage of APP is indeed a specific effect, which is not caused by reduced protein production.

The structure–activity relationship analysis revealed that the effect of lead structure analogs is most prominent with specific residues at C-6 (R^1^) and the exocyclic amino group (R^2^). We found that when R^1^ represents halogens and other electron-withdrawing substituents, for example, nitro, trifluoromethyl and cyano, this leads to a strong increase in mitochondrial membrane potential, while also strongly attenuating Aβ peptide production and ER calcium release. On the other hand, substitution at C-7 and C-8 (gea_84) was found to be detrimental to the activity. Furthermore, *N*-methylation at either the pyrrole nitrogen (gea_90) or the side chain secondary amino group (gea_92) led to the complete loss of activity. Merely aliphatic residues at the exocyclic nitrogen (R^2^; that is, 5781439, 5781448, 5781457, gea_87) result in lowering or loss of the activity, whereas additional attachment of an aromatic motif (phenyl group) shows benefit in all three assays (that is, 5781464, 5781441). The best effect in all the three assays was detectable for tetrahydrocarbazoles containing a diamino side chain R^2^ (4-aminopiperidine) with an attached *N*-benzyl or *N*-phenethyl residue (that is, 5781441, 5781464, gea_96, gea_97, gea_101, gea_102, gea_130, gea_133; Figure 5a). Therefore, by systematic optimization of the primary screening hits, we generated a subclass of compounds that are active in all the three assays.

The vast majority of AD patients are sporadic late-onset cases and age remains the main risk factor for developing sporadic AD.^48^ Importantly, aging process involves disturbances in the intracellular calcium homeostasis, particularly in ER and mitochondria.^49, 50^ Lymphocytes derived from sporadic AD patients show elevated cytosolic basal calcium concentrations.^36, 51^ Every gene that is known to increase susceptibility to AD also modulates some aspect of calcium signaling.^4^ In particular, a polymorphism in the CALHM1 gene encoding an ion channel's pore-forming subunit that affects intracellular calcium homeostasis, has been linked to susceptibility to sporadic AD.^52, 53^ Along with the ER stress, mitochondrial damage also contributes to aging process.^54^ Moreover, sporadic AD is associated with reduced mitochondrial membrane potential,^55^ as well as elevated BACE1 activity,^56^ which, in turn, leads to increased Aβ production and plaque deposition.^57^ Therefore, we predict that the benefits of tetrahydrocarbazoles, will not be limited to familial AD cases, but also may present a high potential for sporadic AD cases as well (patent pending; PCT/EP2013/055969).

The relationship between calcium homeostasis, mitochondrial activity and Aβ formation is rather complex. It is established that the modulation of ER calcium homeostasis can affect mitochondrial function^25^ and APP metabolism.^30^ On the other hand, Aβ pathology and mitochondrial dysfunction can also disrupt intracellular calcium homeostasis.^58, 59^ In addition, Aβ can impair mitochondrial function, and, at the same time, mitochondrial failure can promote Aβ formation.^60^ As the direct molecular target(s) of tetrahydrocarbazoles remains elusive, this study is limited by the fact that we cannot conclude that the improved mitochondrial activity and decreased Aβ production are necessarily downstream effects of normalizing calcium homeostasis. Alternatively, improved mitochondrial function and/or decreased Aβ generation can be upstream of normalized intracellular calcium homeostasis. Therefore, future studies addressing the exact molecular target(s) of tetrahydrocarbazole lead structure and their detailed therapeutic mode of action are of utmost importance. Whether or not the beneficial effects of tetrahydrocarbazoles on calcium homeostasis, mitochondrial function and APP processing follow a dependency, should not, however, impact the therapeutic relevance of this discovery. Another important open question which remains to be elucidated is whether tetrahydrocarbazoles also reverse the late-stage Aβ-plaque-dependent calcium disturbances in the brain.^61, 62^ Apart from IP_3_ receptor channel gating itself,^63^ multiple upstream elements of IP_3_R-mediated calcium release are affected in AD, for example, GPCR in general,^64^ and muscarinic receptors in particular,^65^ G-proteins,^66^ as well as PLC.^67^ Given that tetrahydrocarbazoles may potentially target any of those upstream elements, such a phenotypic multitargeted drug screening assay provides the important advantage of collectively addressing several aspects of AD.
