# Supplementary material for: Identification of tetrahydrocarbazoles as novel multifactorial drug candidates for treatment of Alzheimer's disease
Source: Transl Psychiatry. 2014 Dec 16;4(12):e489–. doi: 10.1038/tp.2014.132 (PMC4270312; doi:10.1038/tp.2014.132)
Supplement: Supplementary Figures [file tp2014132x1.pdf]

Figure S1

Compound Structure and name	ID	Molecular formula	MW	clogP	Calcium	TMRM	Aβ 42	Aβ 40	Aβ 38
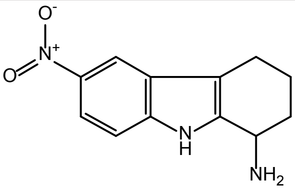 6-nitro-2,3,4,9-tetrahydro-1H-carbazol-1-amine	5302860	$C_{12}H_{13}N_3O_2$	231.25	2.16	1.038	0.622	0.473	0.494	0.421
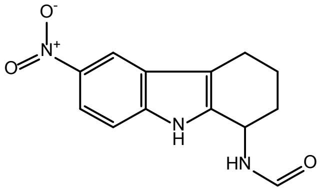 N-(6-nitro-2,3,4,9-tetrahydro-1H-carbazol-1-yl)formamide	5373790	$C_{13}H_{23}N_3O_3$	259.26	1.86	0.944	0.656	0.988	0.875	0.981
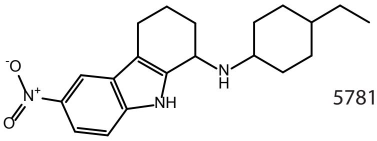 N-(4-ethylcyclohexyl)-6-nitro-2,3,4,9-tetrahydro-1H-carbazol-1-amine	5781439	$C_{20}H_{27}N_3O_2$	341.45	5.65	0.981	1.384	1.058	0.733	0.584
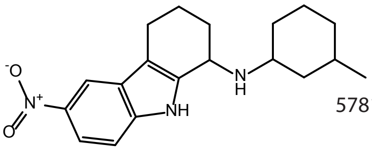 N-(3-methylcyclohexyl)-6-nitro-2,3,4,9-tetrahydro-1H-carbazol-1-amine	5781448	$C_{19}H_{25}N_3O_2$	327.42	5.13	0.966	1.545	0.593	0.726	0.593
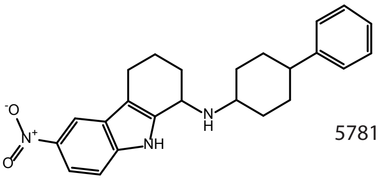 6-nitro-N-(4-phenylcyclohexyl)-2,3,4,9-tetrahydro-1H-carbazol-1-amine	5781451	$C_{24}H_{27}N_3O_2$	389.49	6.01	0.967	1.180	1.093	0.777	0.648
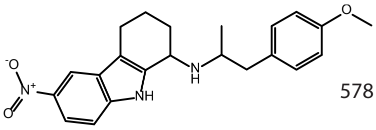 N-(1-(4-methoxyphenyl)propan-2-yl)-6-nitro-2,3,4,9-tetrahydro-1H-carbazol-1-amine	5781452	$C_{22}H_{25}N_3O_3$	379.45	4.90	0.952	2.118	0.961	0.564	0.609
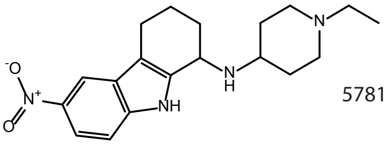 N-(1-ethylpiperidin-4-yl)-6-nitro-2,3,4,9-tetrahydro-1H-carbazol-1-amine	5781457	$C_{19}H_{26}N_4O_2$	342.44	3.16	0.789	1.926	0.702	0.503	0.623
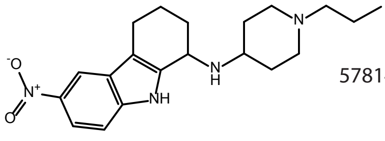 6-nitro-N-(1-propylpiperidin-4-yl)-2,3,4,9-tetrahydro-1H-carbazol-1-amine	5781458	$C_{20}H_{28}N_4O_2$	356.46	3.69	0.962	1.756	0.683	0.503	0.618

Compound Structure and name	ID	Molecular formula	MW	clogP	Calcium	TMRM	Aβ 42	Aβ 40	Aβ 38
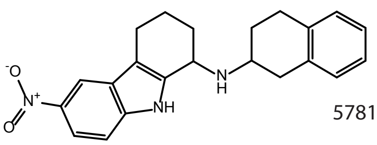 6-nitro-N-(1,2,3,4-tetrahydronaphthalen-2-yl)-2,3,4,9-tetrahydro-1H-carbazol-1-amine	5781459	$C_{22}H_{23}N_3O_2$	361.44	5.12	0.979	1.551	0.980	0.652	0.812
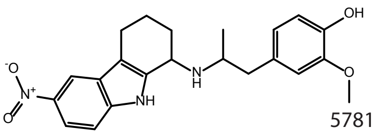 2-methoxy-4-(2-((6-nitro-2,3,4,9-tetrahydro-1H-carbazol-1-yl)amino)propyl)phenol	5781461	$C_{22}H_{25}N_3O_4$	395.45	4.16	1.030	1.499	0.875	0.718	0.794
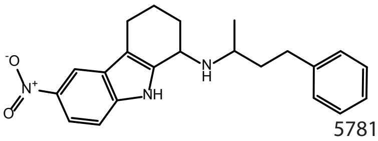 6-nitro-N-(4-phenylbutan-2-yl)-2,3,4,9-tetrahydro-1H-carbazol-1-amine	5781463	$C_{22}H_{25}N_3O_2$	363.45	5.36	0.982	1.128	1.126	0.853	0.807
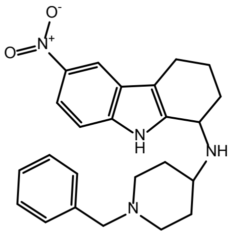 N-(1-benzylpiperidin-4-yl)-6-nitro-2,3,4,9-tetrahydro-1H-carbazol-1-amine	5781464	$C_{24}H_{28}N_4O_2$	404.50	4.42	0.895	3.256	0.575	0.539	0.486
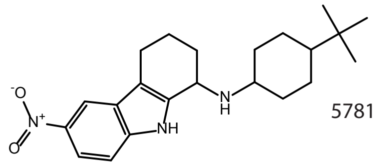 N-(4-(tert-butyl)cyclohexyl)-6-nitro-2,3,4,9-tetrahydro-1H-carbazol-1-amine	5781465	$C_{22}H_{31}N_3O_2$	369.50	6.45	0.959	1.157	0.891	0.747	0.585
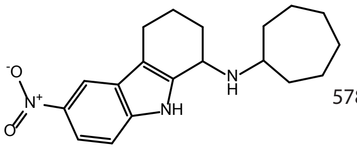 N-cycloheptyl-6-nitro-2,3,4,9-tetrahydro-1H-carbazol-1-amine	5781467	$C_{22}H_{31}N_3O_2$	327.42	5.17	0.923	0.959	1.007	0.785	0.609
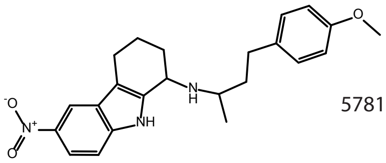 N-(4-(4-methoxyphenyl)butan-2-yl)-6-nitro-2,3,4,9-tetrahydro-1H-carbazol-1-amine	5781468	$C_{23}H_{27}N_3O_3$	393.48	5.28	0.936	1.476	1.000	0.868	0.668
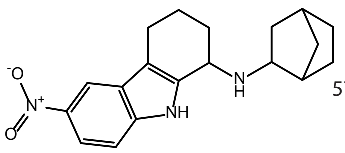 N-(bicyclo[2.2.1]heptan-2-yl)-6-nitro-2,3,4,9-tetrahydro-1H-carbazol-1-amine	5781469	$C_{19}H_{23}N_3O_2$	325.40	4.89	0.942	0.892	1.068	0.596	0.628

Compound Structure and name	ID	Molecular formula	MW	clogP	Calcium	TMRM	Aβ 42	Aβ 40	Aβ 38
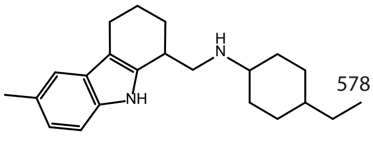 4-ethyl-N-((6-methyl-2,3,4,9-tetrahydro-1H-carbazol-1-yl)methyl)cyclohexanamine	5781474	$C_{22}H_{32}N_2$	324.50	6.21	0.437	0.641	1.471	0.769	0.815
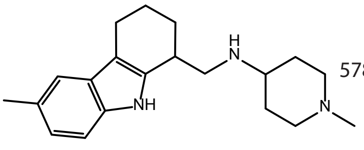 1-methyl-N-((6-methyl-2,3,4,9-tetrahydro-1H-carbazol-1-yl)methyl)piperidin-4-amine	5781475	$C_{20}H_{29}N_3$	311.46	3.19	0.499	1.259	1.068	0.753	0.916
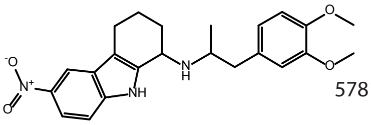 N-(1-(3,4-dimethoxyphenyl)propan-2-yl)-6-nitro-2,3,4,9-tetrahydro-1H-carbazol-1-amine	5781442	$C_{23}H_{27}N_3O_4$	409.48	4.64	0.961	1.329	1.053	0.692	0.772
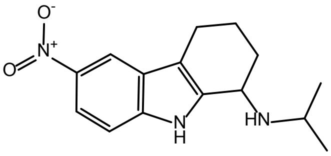 N-isopropyl-6-nitro-2,3,4,9-tetrahydro-1H-carbazol-1-amine	5781470	$C_{15}H_{19}N_3O_2$	273.33	3.41	0.942	0.850	0.760	0.545	0.582
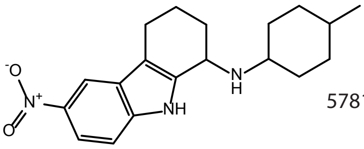 N-(4-methylcyclohexyl)-6-nitro-2,3,4,9-tetrahydro-1H-carbazol-1-amine	5781447	$C_{19}H_{25}N_3O_2$	327.42	5.13	0.942	0.491	0.983	0.827	0.735
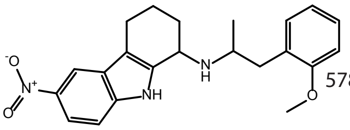 N-(1-(2-methoxyphenyl)propan-2-yl)-6-nitro-2,3,4,9-tetrahydro-1H-carbazol-1-amine	5781453	$C_{22}H_{25}N_3O_3$	379.45	4.90	0.917	0.554	0.896	0.779	0.850
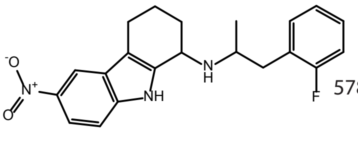 N-(1-(2-fluorophenyl)propan-2-yl)-6-nitro-2,3,4,9-tetrahydro-1H-carbazol-1-amine	5781454	$C_{21}H_{22}FN_3O_2$	367.42	5.12	0.925	0.433	0.846	0.706	0.692
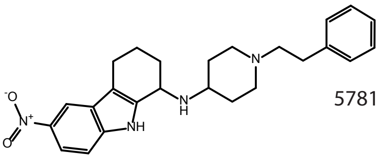 6-nitro-N-(1-phenethylpiperidin-4-yl)-2,3,4,9-tetrahydro-1H-carbazol-1-amine	5781441	$C_{25}H_{30}N_4O_2$	418.53	4.56	0.914	2.357	0.566	0.531	0.523

Compound Structure and name	ID	Molecular formula	MW	clogP	Calcium	TMRM	Aβ 42	Aβ 40	Aβ 38
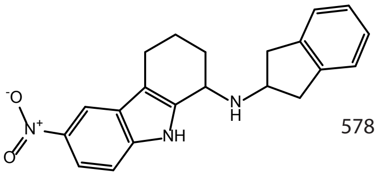 N-(2,3-dihydro-1H-inden-2-yl)-6-nitro-2,3,4,9-tetrahydro-1H-carbazol-1-amine	5781444	$C_{21}H_{21}N_3O_2$	347.41	4.56	0.917	1.046	0.785	0.769	0.857
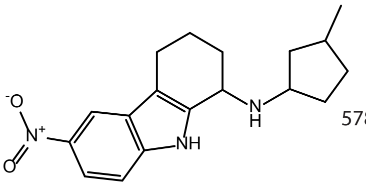 1-methyl-N-((6-methyl-2,3,4,9-tetrahydro-1H-carbazol-1-yl)methyl)piperidin-4-amine	5781446	$C_{18}H_{23}N_3O_2$	313.39	4.57	0.933	1.259	1.038	0.593	0.594
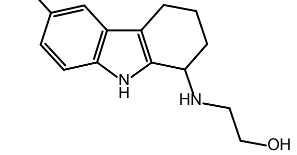 2-((6-bromo-2,3,4,9-tetrahydro-1H-carbazol-1-yl)amino)ethanol	5258947	$C_{14}H_{17}BrN_2O$	309.20	2.91	1.047	1.514	0.335	0.355	0.317
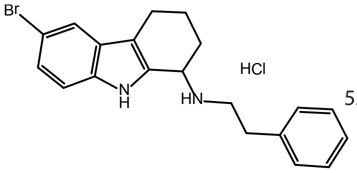 6-bromo-N-phenethyl-2,3,4,9-tetrahydro-1H-carbazol-1-amine hydrochloride	5265927	$C_{20}H_{22}BrClN_2$	405.76	5.60	0.791	1.922	0.292	0.278	0.176
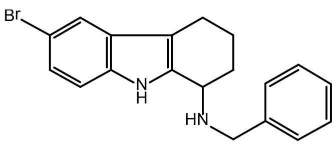 N-benzyl-6-bromo-2,3,4,9-tetrahydro-1H-carbazol-1-amine	5307331	$C_{19}H_{19}BrN_2$	355.27	4.66	0.703	1.508	0.683	0.503	0.357
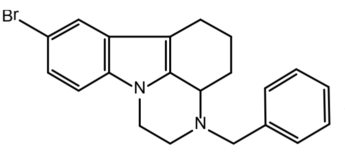 3-benzyl-8-bromo-2,3,3a,4,5,6-hexahydro-1H-pyrazino[3,2,1-jk]carbazole	5358447	$C_{21}H_{21}BrN_2$	381.31	6.11	0.953	1.008	0.332	0.397	0.421
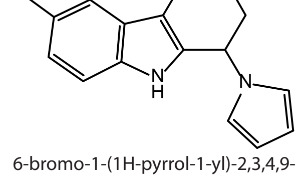 6-bromo-1-(1H-pyrrol-1-yl)-2,3,4,9-tetrahydro-1H-carbazole	5920301	$C_{16}H_{15}BrN_2$	315.21	4.98	0.869	0.831	0.492	0.481	0.449
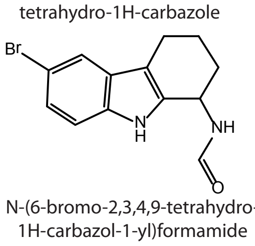 N-(6-bromo-2,3,4,9-tetrahydro-1H-carbazol-1-yl)formamide	5922537	$C_{13}H_{13}BrN_2O$	293.16	2.79	1.002	1.207	1.056	0.712	0.874

Compound Structure and name	ID	Molecular formula	MW	clogP	Calcium	TMRM	Aβ 42	Aβ 40	Aβ 38
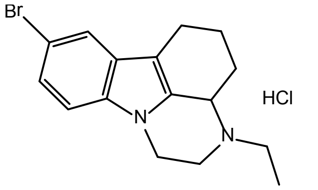 8-bromo-3-ethyl-2,3,3a,4,5,6-hexahydro-1H-pyrazino[3,2,1-jk]carbazole hydrochloride	6044730	$C_{16}H_{20}BrClN_2$	355.70	4.72	0.968	0.745	1.180	0.745	1.003
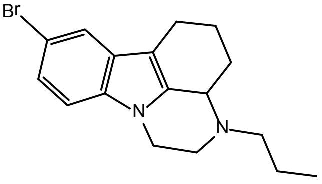 8-bromo-3-propyl-2,3,3a,4,5,6-hexahydro-1H-pyrazino[3,2,1-jk]carbazole	6049723	$C_{17}H_{21}BrN_2$	333.27	5.25	0.961	0.663	0.840	0.642	0.817
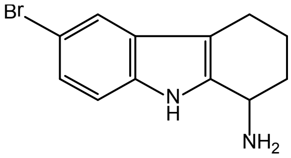 6-bromo-2,3,4,9-tetrahydro-1H-carbazol-1-amine	5303801	$C_{12}H_{13}BrN_2$	265.15	3.09	0.989	1.116	0.269	0.271	0.309
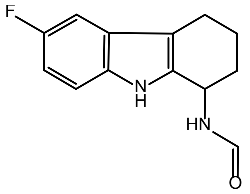 N-(6-fluoro-2,3,4,9-tetrahydro-1H-carbazol-1-yl)formamide	7129409	$C_{13}H_{13}FN_2O$	232.25	2.07	0.958	0.860	0.592	0.470	0.589
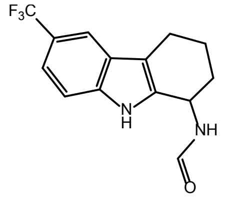 N-(6-(trifluoromethyl)-2,3,4,9-tetrahydro-1H-carbazol-1-yl)formamide	5922756	$C_{14}H_{13}F_3N_2O$	282.26	2.93	1.001	1.236	0.999	1.033	1.057
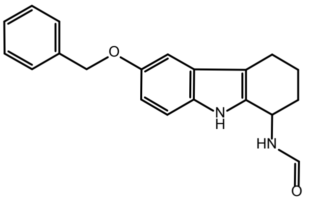 N-(6-(benzyloxy)-2,3,4,9-tetrahydro-1H-carbazol-1-yl)formamide	7173317	$C_{20}H_{20}N_2O_2$	320.38	3.55	0.998	0.606	1.012	0.997	1.104

Figure S2

Compound Structure and name	ID	Molecular formula	MW	clogP	Calcium	TMRM	Aβ 42	Aβ 40	Aβ 38
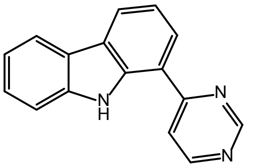 1-(pyrimidin-4-yl)-9H-carbazole	gea_82	$C_{16}H_{11}N_3$	245.28	3.16	0.974	0.926	1.080	1.086	1.164
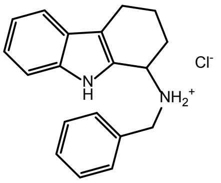 N-benzyl-2,3,4,9-tetrahydro-1H-carbazol-1-aminium chloride	gea_83	$C_{19}H_{28}ClN_2$	312.84	4.63	0.991	1.219	1.050	0.835	0.635
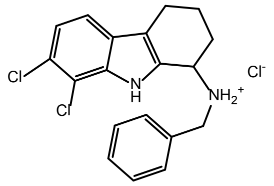 N-benzyl-7,8-dichloro-2,3,4,9-tetrahydro-1H-carbazol-1-aminium chloride	gea_84	$C_{19}H_{19}Cl_3N_2$	381.73	6.16	1.011	1.049	1.100	1.050	0.913
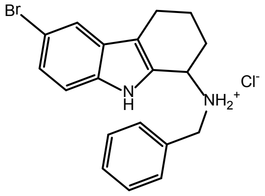 N-benzyl-6-bromo-2,3,4,9-tetrahydro-1H-carbazol-1-aminium chloride	gea_85	$C_{19}H_{20}BrClN_2$	391.73	5.66	0.879	1.444	0.910	0.765	0.651
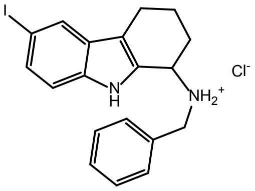 N-benzyl-6-iodo-2,3,4,9-tetrahydro-1H-carbazol-1-aminium chloride	gea_86	$C_{19}H_{20}ClIN_2$	438.73	5.92	0.913	1.444	0.901	0.767	0.629
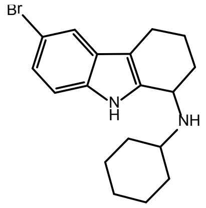 6-bromo-N-cyclohexyl-2,3,4,9-tetrahydro-1H-carbazol-1-amine	gea_87	$C_{18}H_{23}BrN_2$	347.29	5.53	0.946	0.998	0.779	0.564	0.479
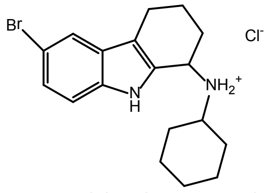 6-bromo-N-cyclohexyl-2,3,4,9-tetrahydro-1H-carbazol-1-aminium chloride	gea_87_HCl	$C_{18}H_{24}BrClN_2$	383.75	6.01	0.914	0.926	0.848	0.608	0.610
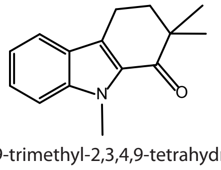 2,2,9-trimethyl-2,3,4,9-tetrahydro-1H-carbazol-1-one	gea_88	$C_{15}H_{17}NO$	227.30	3.80	0.977	0.814	1.218	0.850	1.013

Compound Structure and name	ID	Molecular formula	MW	clogP	Calcium	TMRM	Aβ 42	Aβ 40	Aβ 38
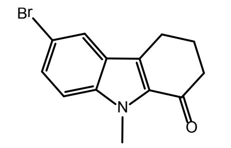 6-bromo-9-methyl-2,3,4,9-tetrahydro-1H-carbazol-1-one	gea_89	$C_{13}H_{12}BrNO$	278.14	3.65	0.947	0.858	0.956	0.772	0.948
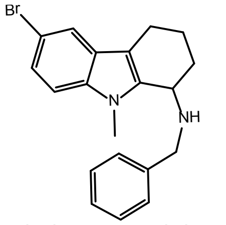 N-benzyl-6-bromo-9-methyl-2,3,4,9-tetrahydro-1H-carbazol-1-amine	gea_90	$C_{20}H_{21}BrN_2$	369.30	5.00	0.972	1.024	0.891	0.806	0.892
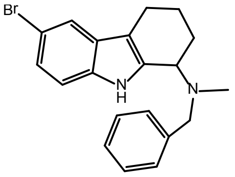 N-benzyl-6-bromo-N-methyl-2,3,4,9-tetrahydro-1H-carbazol-1-amine	gea_92	$C_{20}H_{21}BrN_2$	369.30	5.74	1.000	0.724	0.815	0.754	0.745
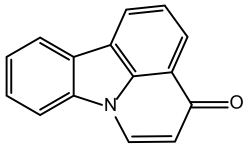 4H-pyrido[3,2,1-jk]carbazol-4-one	gea_94	$C_{15}H_9NO$	219.24	1.96	0.960	0.716	1.075	1.110	1.019
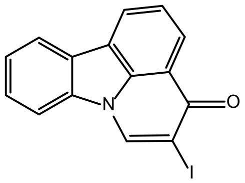 5-iodo-4H-pyrido[3,2,1-jk]carbazol-4-one	gea_95	$C_{15}H_8INO$	345.13	3.09	0.974	0.926	1.031	0.993	0.991
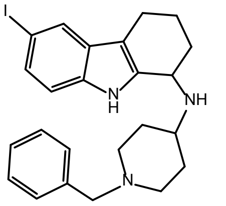 N-(1-benzylpiperidin-4-yl)-6-iodo-2,3,4,9-tetrahydro-1H-carbazol-1-amine	gea_96	$C_{24}H_{28}IN_3$	485.40	5.61	0.840	2.100	0.542	0.520	0.461
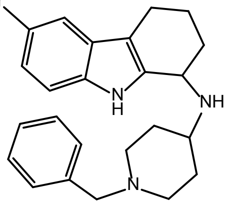 N-(1-benzylpiperidin-4-yl)-6-bromo-2,3,4,9-tetrahydro-1H-carbazol-1-amine	gea_97	$C_{24}H_{28}BrN_3$	438.40	5.35	0.894	2.335	0.530	0.552	0.487
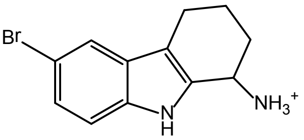 6-bromo-2,3,4,9-tetrahydro-1H-carbazol-1-aminium	gea_99	$C_{12}H_{14}BrClN_2$	301.61	3.14	0.997	1.563	0.506	0.489	0.586

Compound Structure and name	ID	Molecular formula	MW	clogP	Calcium	TMRM	Aβ 42	Aβ 40	Aβ 38
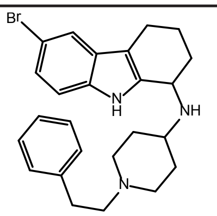 6-bromo-N-(1-phenethylpiperidin-4-yl)-2,3,4,9-tetrahydro-1H-carbazol-1-amine	gea_101	$C_{25}H_{30}BrN_3$	452.43	5.49	0.856	1.929	0.449	0.513	0.441
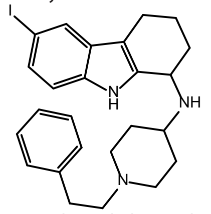 6-iodo-N-(1-phenethylpiperidin-4-yl)-2,3,4,9-tetrahydro-1H-carbazol-1-amine	gea_102	$C_{25}H_{30}IN_3$	499.43	5.74	0.871	1.726	0.523	0.480	0.479
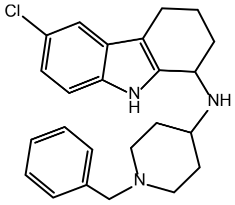 N-(1-benzylpiperidin-4-yl)-6-chloro-2,3,4,9-tetrahydro-1H-carbazol-1-amine	gea_129	$C_{24}H_{28}ClN_3$	393.95	5.2	0.832	1.862	0.327	0.383	0.311
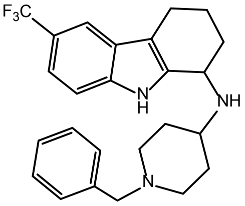 N-(1-benzylpiperidin-4-yl)-6-(trifluoromethyl)-2,3,4,9-tetrahydro-1H-carbazol-1-amine	gea_130	$C_{25}H_{28}F_3N_3$	427.51	5.50	0.763	2.214	0.426	0.478	0.514
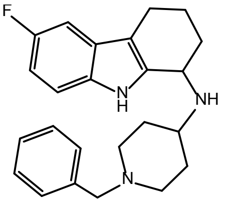 N-(1-benzylpiperidin-4-yl)-6-fluoro-2,3,4,9-tetrahydro-1H-carbazol-1-amine	gea_132	$C_{24}H_{28}FN_3$	377.50	4.63	0.834	2.004	0.295	0.361	0.342
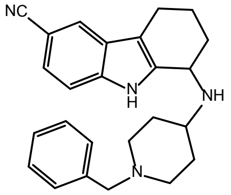 1-((1-benzylpiperidin-4-yl)amino)-2,3,4,9-tetrahydro-1H-carbazole-6-carbonitrile	gea_133	$C_{25}H_{28}N_4$	384.52	4.14	0.899	2.636	0.273	0.308	0.296
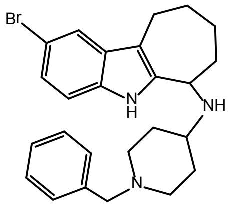 N-(1-benzylpiperidin-4-yl)-2-bromo-5,6,7,8,9,10-hexahydrocyclohepta[b]indol-6-amine	gea_139	$C_{25}H_{30}BrN_3$	452.43	5.91	0.963	1.646	0.504	0.576	0.514

Figure S3

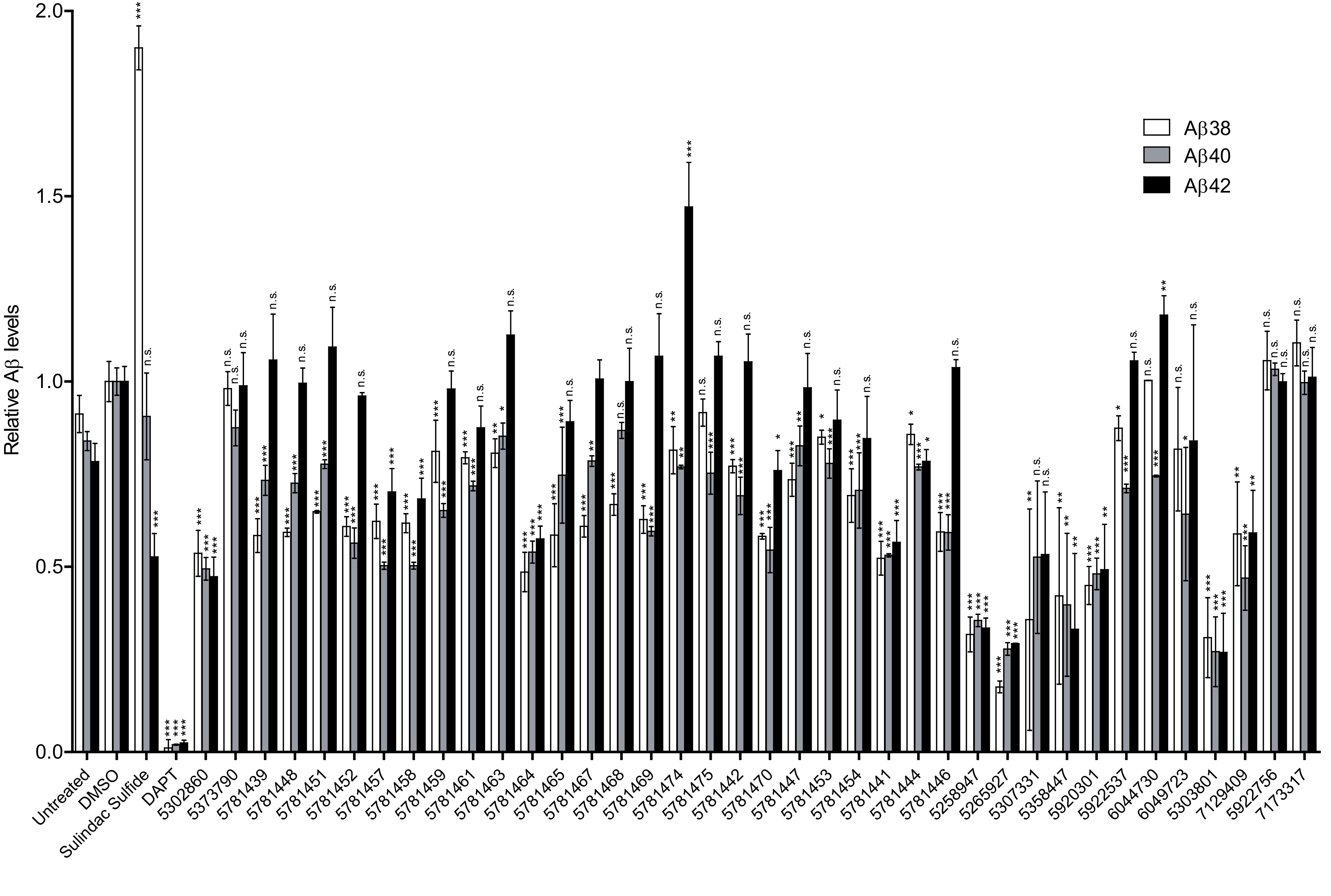

Relative Aβ levels
0.0
0.5
1.0
1.5
2.0
Aβ38
Aβ40
Aβ42
Untreated
DMSO
Sulindac Sulfide
DAPT
5302860
5373790
5781439
5781448
5781451
5781452
5781457
5781458
5781459
5781461
5781463
5781464
5781465
5781467
5781468
5781469
5781474
5781475
5781442
5781470
5781447
5781453
5781454
5781441
5781444
5781446
5258947
5265927
5307331
5358447
5920301
5922537
6044730
6049723
5303801
7129409
5922756
7173317

Figure S4

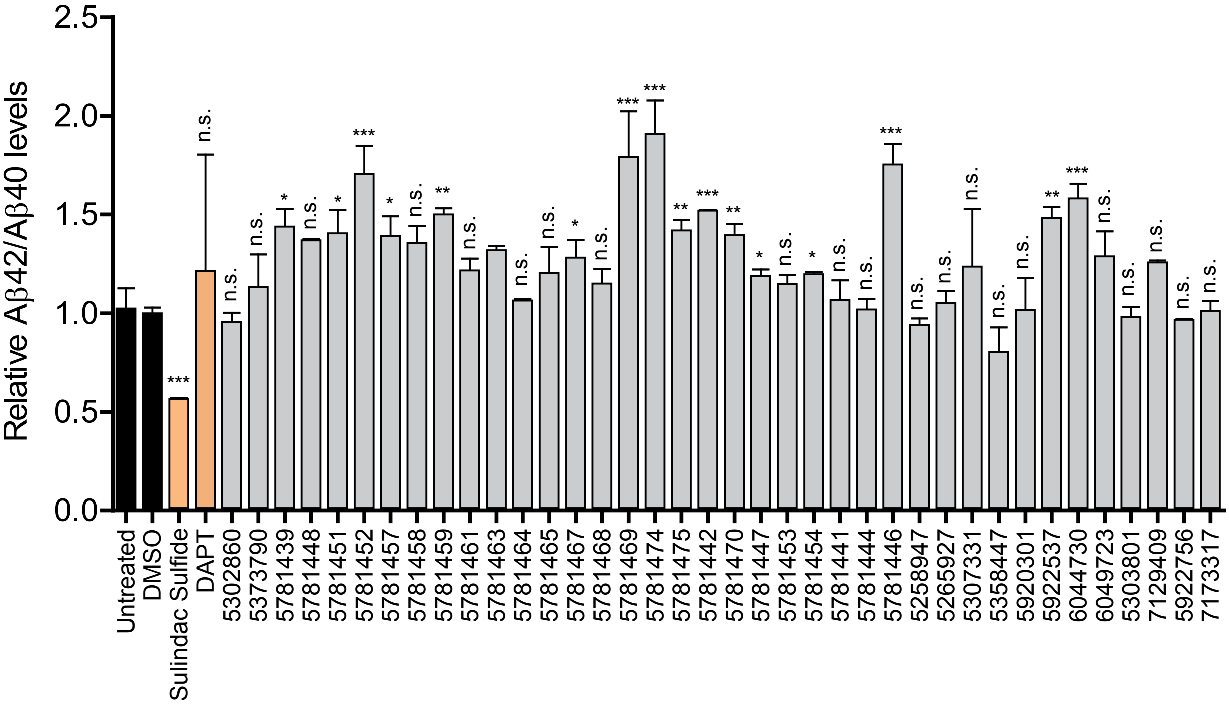

Relative Aβ42/Aβ40 levels
2.5
2.0
1.5
1.0
0.5
0.0
Untreated
DMSO
Sulindac Sulfide
DAPT
5302860
5373790
5781439
5781448
5781451
5781452
5781457
5781458
5781459
5781461
5781463
5781464
5781465
5781467
5781468
5781469
5781474
5781475
5781442
5781470
5781447
5781453
5781454
5781441
5781444
5781446
5258947
5265927
5307331
5358447
5920301
5922537
6044730
6049723
5303801
7129409
5922756
7173317
n.s.
*
**
***
