# Supplementary material for: Identification of tetrahydrocarbazoles as novel multifactorial drug candidates for treatment of Alzheimer's disease
Source: Transl Psychiatry. 2014 Dec 16;4(12):e489–. doi: 10.1038/tp.2014.132 (PMC4270312; doi:10.1038/tp.2014.132)
Supplement: Supplementary Information [file tp2014132x2.doc]

**Supporting information**

Identification of tetrahydrocarbazoles as novel multifactorial drug candidates in the treatment of Alzheimer’s disease

Kamran Honarnejad, Alexander Daschner , André P. Gehring, Aleksandra Szybinska, Armin Giese, Jacek Kuznicki, Franz Bracher and Jochen Herms

**Supplementary figure legends**

*Fig. S1. The list of commercially available tetrahydrocarbazole analogues*

Shown are 38 tested commercially available tetrahydrocarbazoles. Next to their chemical structure and physical properties, the measures for their activity in different assays are presented with their corresponding normalized values for: CCh-evoked calcium release peak; mitochondrial membrane potential (TMRM); and the levels of three different secreted Aβ peptides (measured at 10 µM).

*Fig. S2. The list of synthesized tetrahydrocarbazoles and analogues*

Shown are 23 strategically synthesized tetrahydrocarbazole analogues. Next to their chemical structure and physical properties, the measures for their activity in different assays are presented with their corresponding normalized values for: CCh-evoked calcium release peak; mitochondrial membrane potential (TMRM); and the levels of three different secreted Aβ peptides (measured at 10 µM).

*Fig. S3. The effect of commercially available tetrahydrocarbazole analogues on Aβ production*

Relative Aβ38, Aβ40 and Aβ42 levels are decreased after 16 h treatment with commercially available tetrahydrocarbazole analogues at 10 µM in HEK293 cells coexpressing APPsw and PS1-M146L. Sulindac sulfide (50 µM) and DAPT (10 µM), respectively, a γ-secretase modulator and a γ-secretase inhibitor, were used as positive controls. All the values are normalized to the value of DMSO, which is set to 1. (n.s.: non-significant; * P<0.05, ** P<0.01 and *** P<0.001; n=2).

*Fig. S4. The effect of commercially available tetrahydrocarbazole analogues on Aβ42/Aβ40 ratio*

Relative Aβ42/Aβ40 ratios calculated from figure S2. Treatment with the majority of the lead structure derivatives does not alter the Aβ42/Aβ40 ratio, whereas the positive control Sulindac sulfide (but not DAPT), significantly lowers the Aβ42/Aβ40 ratio. All values are normalized to the value of DMSO, which is set to 1. (n.s.: non-significant; * P<0.05, ** P<0.01 and *** P<0.001; n=2).

Supplementary Materials and Methods

*Commercially available compound library and derivatives:* The DIVERSet® compound library and further commercially available tetrahydrocarbazole analogues and related structures were obtained from ChemBridge (ChemBridge Corp., San Diego, CA). The database for the library compounds and the tested analogues are available at [http://www.chembridge.com](http://www.chembridge.com/) and [https://www.hit2lead.com](https://www.hit2lead.com/)

*Synthesis of the derivative compounds:* The target **2,3,4,9-tetrahydro-1*H*-carbazol-1-amines** were prepared in 1 step from appropriately substituted 2,3,4,9-tetrahydro-1*H*-carbazol-1-ones by reductive amination with primary amines (or ammonia for **gea_99**) using the sodium borohydride/magnesium perchlorate reagent, sodium triacetoxyborohydride or sodium cyanoborohydride. The products were isolated as free amines or as hydrochlorides.

Ring homologue **gea_139** was obtained in an analogous manner from the seven-membered ketone analogue.

Tertiary amine **gea_92** was obtained by *N*-methylation of **gea_85**, ketones **gea_88**  and **gea_89** (as a precursor of N^9^-methylated 1-aminotetrahydrocarbazole **90**) by methylation of appropriate 2,3,4,9-tetrahydro-1*H*-carbazol-1-ones.

**General Information:** NMR spectra were recorded using a Jeol JNMR‑GSX 400 or Jeol JNMR‑GSX 500 (Jeol, Peabody, USA), chemical shifts are given in ppm, coupling constants in Hertz. Mass spectra (electronic ionization, EI, 70 eV) were recorded using a Hewlett Packard 5989 A Mass Spectrometer with a 59980 B Particle Beam LC/MS-interface (Agilent Technologies, Palo Alto, USA). High resolution mass spectra were obtained using a Jeol JMS GCmate II (Jeol, Peabody, USA). IR spectra were recorded as KBr discs on a PerkinElmer FT‑IR Paragon 1000 (PerkinElmer, Waltham, USA) or Jasco FT/IR‑410 (Jasco, Easton, USA). Melting points were determined with a Büchi B‑540 apparatus (Büchi, Flawil, Switzerland) and are uncorrected. Purification of products by flash column chromatography (FCC) was done using Silica gel 60 (Merck, Darmstadt, Germany). HPLC purities were obtained using a HP Agilent 1100 HPLC equipped with a diode array detector (Agilent Technologies, Waldbronn, Germany) and an Agilent Poroshell column (120 EC-C18, 3.0 x 100 mm, 2.7 Micron). Mobile phase consisted of acetonitrile/water/THF/1M NaOH (850:150:1.5:0.3), flow rate was set at 0.7 mL/min at a constant temperature of 45 °C. The chromatographic separations were monitored at 254 nm, using a band width of 4 nm. Injection volume was 10 µL of a dilution of 100 µg/mL (sample in mobile phase).

All chemicals were purchased from Sigma-Aldrich, Fluka and Acros.

**Tetrahydrocarbazoles:**

**(±)-*N^1^*-Benzyl-2,3,4,9-tetrahydro-1*H*-carbazol-1-amine hydrochloride (gea_83)**

C_19_H_21_ClN_2_; M*_r_* = 312.84 g/mol (free amine: C_19_H_20_N_2_; M*_r_* = 276.38 g/mol)

A suspension of 200 mg (1.08 mmol) 2,3,4,9-tetrahydro-1*H*-carbazol-1-one [^1^](#_ENREF_1), 12 mg (0.054 mmol) magnesium perchlorate, and 286 µL (2.62 mmol) benzylamine in 20 mL 1,2-dichlorethane is stirred at room temperature for 8 h, then concentrated to dryness. The residue is dissolved in 20 mL methanol, treated with 78 mg (2.1 mmol) sodium borohydride and stirred for 5 h. Then 20 mL of a saturated sodium bicarbonate solution are added, and the mixture is extracted with ethyl acetate (3 x 15 mL). The combined organic layers are dried over sodium sulfate, concentrated, and the residue purified by FCC (isohexane/ethyl acetate 2:1). The pure product is dissolved in diethyl ether, and HCl gas is passed through the solution until precipitation ceases. The precipitate is collected and dried in vacuo.

**Yield:** 180 mg (54 %), white powder

**Melting range:** 187 – 189 °C

**^1^H-NMR (500 MHz, DMSO-d_6_):** δ (ppm) = 11.46 (br s, 1H, 9-NH), 9.95 (br s, 1H, 1´-NH), 9.80 (br s, 1H, 1´-NH), 7.74 – 7.63 (m, 2H, 2´´-H, 6´´-H), 7.49 (d, *J* = 7.6 Hz, 1H, 5-H), 7.45 – 7.38 (m, 4H, 8-H, 3´´-H, 4´´-H, 5´´-H), 7.15 (t, *J* = 7.6 Hz, 1H, 7-H), 7.02 (t, *J* = 7.5 Hz, 1H, 6-H), 4.68 (s, 1H, 1-H), 4.27 (t, *J* = 6.1 Hz, 2H, 1´-H), 2.77 – 2.60 (m, 2H, 4-H), 2.28 – 2.20 (m, 2H, 2-H), 2.15 – 2.06 (m, 1H, 3-H), 1.85 – 1.74 (m, 1H, 3-H).

**^13^C-NMR (100 MHz, DMSO-d_6_):** δ (ppm) = 135.9 (C-8a), 131.9 (C-1´´), 130.1 (C-2´´, C-6´´), 128.7 (C-4´´), 128.4 (C-3´´, C-5´´), 127.5 (C-9a), 125.9 (C-4b), 122.3 (C-7), 118.8 (C-6), 118.5 (C-5), 113.7 (C-4a), 111.3 (C-8), 50.9 (C-1), 47.5 (C-1´), 25.3 (C-2), 20.2 (C-4), 19.8 (C-3).

**IR (KBr):** $\tilde{\nu}$ (cm^-1^) = 3425, 3257, 2929, 2754, 2577, 2436, 1624, 1586, 1498, 1455, 1438, 1411, 1358, 1330, 1305, 1226, 1156, 1015, 975, 922, 752, 737, 694, 665, 599, 582, 569, 532, 500, 486

**MS (CI):** *m/z* (rel. int. in %) = 277 [M^+^ + H] (50), 170 (100), 108 (10)

**MS (EI):** *m/z* (rel. int. in %) = 276 [M^+^**^•^**] (35), 248 (15), 185 (15), 169 (100), 157 (40), 157 (40), 143 (10), 130 (10), 106 (15), 91 (40), 77 (10), 65 (15)

**HR-MS (EI):** *m/z* = 276.1632 (calculated for C_19_H_20_N_2_: 276.1627)

**HPLC purity:** > 99 %

**(±)-*N^1^*-Benzyl-7,8-dichloro-2,3,4,9-tetrahydro-1*H*-carbazol-1-amine hydrochloride (gea_84)**

C_19_H_19_Cl_3_N_2_; M*_r_* = 381.73 g/mol (free amine: C_19_H_18_Cl_2_N_2_; M*_r_* = 345.27 g/mol)

A suspension of 200 mg (0.787 mmol) 7,8-dichloro-2,3,4,9-tetrahydro-1*H*-carbazol-1-one [^2^](#_ENREF_2), 9 mg (0.04 mmol) magnesium perchlorate, and 205 mg (1.92 mmol) benzylamine in 20 mL 1,2-dichlorethane is stirred at room temperature for 8 h, then concentrated to dryness. The residue is dissolved in 20 mL methanol, treated with 57 mg (1.5 mmol) sodium borohydride and stirred for 5 h. Then 20 mL of a saturated sodium bicarbonate solution are added, and the mixture is extracted with ethyl acetate (3 x 15 mL). The combined organic layers are dried over sodium sulfate, concentrated, and the residue purified by FCC (dichloromethane/ethyl acetate/ triethylamine 9:1:0.5). The pure product is dissolved in diethyl ether, and HCl gas is passed through the solution until precipitation ceases. The precipitate is collected and dried in vacuo.

**Yield:** 125 mg (42 %), pale brown solid

**Melting range:** 165 – 167 °C

**^1^H-NMR (500 MHz, DMSO-d_6_):** δ (ppm) = 11.89 (br s, 1H, 9-NH), 10.12 (br s, 1H, 1´-NH), 9.92 (br s, 1H, 1´-NH), 7.68 (d, *J* = 6.5 Hz, 2H, 2´´-H, 6´´-H), 7.49 (d, *J* = 8.4 Hz, 1H, 5-H), 7.42 (m, 3H, 3´´-H, 4´´-H, 5´´-H), 7.22 (d, *J* = 8.4 Hz, 1H, 6-H), 4.74 (s, 1H, 1-H), 4.26 (m, 2H, 1´H), 2.68 (t, *J* = 5.4 Hz, 2H, 4-H), 2.37 – 2.25 (m, 1H, 2-H), 2.24 – 2.15 (m, 1H, 2-H), 2.15 – 2.03 (m, 1H, 3-H), 1.84 – 1.70 (m, 1H, 3-H).

**^13^C-NMR (125 MHz, DMSO-d_6_):** δ (ppm) = 134.0 (C-8a), 132.0 (C-1´´), 130.3 (C-2´´, C-6´´), 130.2 (C-9a), 128.9 (C-4´´), 128.6 (C-3´´, C-5´´), 126.3 (C-4b), 124.6 (C-7), 120.8 (C-6), 118.7 (C-5), 115.6 (C-4a), 114.2 (C-8), 50.8 (C-1), 47.3 (C-1´), 24.9 (C-2), 20.2 (C-4), 20.1 (C-3).

**IR (KBr):** $\tilde{\nu}$ (cm^-1^) = 3424, 3221, 2938, 2782, 2417, 2363, 1618, 1575, 1499, 1456, 1443, 1361, 1323, 1225, 1157, 1122, 972, 913, 793, 745, 696, 579, 556, 488

**MS (CI):** *m/z* (rel. int. in %) = 347 [M^+^ + H] (25), 345 (15), 239 (100), 198 (30), 120 (10), 108 (25)

**MS (EI):** *m/z* (rel. int. in %) = 349 (2), 347 (25), 345 [M^+^**^•^**] (15), 317 (25), 254 (15), 242 (3), 240 (40), 238 (55), 226 (25), 202 (20), 190 (20), 167 (10), 106 (40), 91 (100), 77 (10), 65 (20), 51 (10)

**HR-MS (EI):** *m/z* = 344.0865 (calculated for C_19_H_18_Cl_2_N_2_: 344.0847)

**HPLC purity:** 99 %

**(±)-*N^1^*-Benzyl-6-bromo-2,3,4,9-tetrahydro-1*H*-carbazol-1-amine hydrochloride (gea_85)**

C_19_H_20_BrClN_2_;M*_r_* = 391.73 g/mol (free amine: C_19_H_19_BrN_2_; M*_r_* = 355.27 g/mol)

A suspension of 209 mg (0.791 mmol) 6-bromo-2,3,4,9-tetrahydro-1*H*-carbazol-1-one[^3^](#_ENREF_3), 9 mg (0.04 mmol) magnesium perchlorate, and 205 mg (1.92 mmol) benzylamine in 20 mL 1,2-dichlorethane is stirred at room temperature for 8 h, then concentrated to dryness. The residue is dissolved in 20 mL methanol, treated with 57 mg (1.5 mmol) sodium borohydride and stirred for 5 h. Then 20 mL of a saturated sodium bicarbonate solution are added, and the mixture is extracted with ethyl acetate (3 x 15 mL). The combined organic layers are dried over sodium sulfate, concentrated, and the residue purified by FCC (isohexan/ethyl acetate 2:1). The pure product is dissolved in diethyl ether, and HCl gas is passed through the solution until precipitation ceases. The precipitate is collected and dried in vacuo.

**Yield:** 70 mg (23 %), white solid

**Melting range:** 204 – 206 °C (ref. [^4^](#_ENREF_4): no melting range given)

**^1^H-NMR (500 MHz, DMSO-d_6_):** δ (ppm) = 11.69 (br s, 1H, 9-NH), 9.94 (br s, 1H, 1´-NH), 9.80 (br s, 1H, 1´-NH), 7.73 – 7.61 (m, 3H, 5-H, 2´´-H, 6´´-H), 7.47 – 7.36 (m, 4H, 8-H, 3´´-H, 4´´-H, 5´´-H), 7.25 (dd, *J* = 8.6 Hz, 1.8 Hz, 1H, 7-H), 4.68 (s, 1H, 1-H), 4.35 – 4.21 (m, 2H, 1´-H), 2.75 – 2.59 (m, 2H, 4-H), 2.28 – 2.18 (m, 2H, 2-H), 2.16 – 2.02 (m, 1H, 3-H), 1.85 – 1.71 (m, 1H, 3-H).

**^13^C-NMR (100 MHz, DMSO-d_6_):** δ (ppm) = 134.5 (C-8a), 131.8 (C-1´´), 130.1 (C-2´´, C-6´´), 129.3 (C-9a), 128.8 (C-4´´), 128.4 (C-3´´, C-5´´), 127.7 (C-4b), 124.7 (C-7), 120.9 (C-5), 113.5 (C-4a), 113.4 (C-8), 111.3 (C-6), 50.8 (C-1), 47.6 (C-1´), 25.2 (C-2), 20.0 (C-4), 19.5 (C-3).

**IR (KBr):** $\tilde{\nu}$ (cm^-1^) = 3424, 3228, 2938, 2754, 2578, 2411, 1629, 1589, 1560, 1499, 1447, 1409, 1317, 1278, 1238, 1226, 1151, 1049, 1015, 973, 913, 891, 855, 802, 749, 696, 669, 640, 596, 539, 487

**MS (CI):** *m/z* (rel. int. in %) = 357 [M^+^ + H] (85), 355 (100), 276 (20), 250 (100), 248 (85), 169 (10), 120 (10), 108 (45)

**MS (EI):** *m/z* (rel. int. in %) = 356 [M^+^**^•^**] (35), 354 (35), 328 (30), 326 (30), 249 (100), 247 (90), 237 (20), 235 (20), 168 (70), 106 (15), 91 (65), 65 (10)

**HR-MS (EI):** *m/z* = 354.0747 (calculated for C_19_H_19_BrN_2_: 354.0732)

**HPLC purity:** > 99 %

**(±)-*N^1^*-Benzyl-6-iodo-2,3,4,9-tetrahydro-1*H*-carbazol-1-amine hydrochloride (gea_86)**

C_19_H_20_ClIN_2_; M*_r_* = 438.73 g/mol (free amine: C_19_H_19_IN_2_; M*_r_* = 402.27 g/mol)

A suspension of 246 mg (0.791 mmol) 6-iodo-2,3,4,9-tetrahydro-1*H*-carbazol-1-one[^5^](#_ENREF_5), 9 mg (0.04 mmol) magnesium perchlorate, and 205 mg (1.92 mmol) benzylamine in 20 mL 1,2-dichlorethane is stirred at room temperature for 8 h, then concentrated to dryness. the residue is dissolved in 20 mL methanol, treated with 57 mg (1.5 mmol) sodium borohydride and stirred for 5 h. Then 20 mL of a saturated sodium bicarbonate solution are added, and the mixture is extracted with ethyl acetate (3 x 15 mL). The combined organic layers are dried over sodium sulfate, concentrated, and the residue purified by FCC (dichloromethane/ethyl acetate 2:1). The pure product is dissolved in diethyl ether, and HCl gas is passed through the solution until precipitation ceases. The precipitate is collected and dried in vacuo.

**Yield:** 146 mg (42 %), pale yellow solid

**Melting range:** 191 – 193 °C (dec.)

**^1^H-NMR (500 MHz, DMSO-d_6_):** δ (ppm) = 11.62 (br s, 1H, 9-NH), 9.89 (br s, 1H, 1´-NH), 9.75 (br s, 1H, 1´-NH), 7.86 (s, 1H, 5-H), 7.67 (d, *J* = 7.2 Hz, 2H, 2´´-H, 6´´-H), 7.48 – 7.35 (m, 4H, 7-H, 3´´-H, 4´´-H, 5´´-H), 7.28 (d, *J* = 8.5 Hz, 1H, 8-H), 4.67 (s, 1H, 1-H), 4.36 – 4.20 (m, 2H, 1´-H), 2.76 – 2.60 (m, 2H, 4-H), 2.28 – 2.16 (m, 2H, 2-H), 2.16 – 2.04 (m, 1H, 3-H), 1.86 – 1.72 (m, 1H, 3-H).

**^13^C-NMR (100 MHz, DMSO-d_6_):** δ (ppm) = 134.9 (C-8a), 131.8 (C-3´), 130.1 (C-7, C-2´´, C-6´´), 128.8 (C-9a, C-4´´), 128.6 (C-4b), 128.4 (C-3´´, C-5´´), 127.0 (C-5), 113.9 (C-8), 113.2 (C-4a), 82.4 (C-6), 50.7 (C-1), 47.6 (C-2´), 25.2 (C-2), 20.0 (C-4), 19.5 (C-3).

**IR (KBr):** $\tilde{\nu}$ (cm^-1^) = 3424, 3238, 2934, 2752, 2577, 2409, 2361, 1629, 1589, 1498, 1455, 1441, 1408, 1313, 1276, 1226, 1194, 1150, 1041, 1014, 972, 888, 867, 799, 758, 747, 695, 668, 630, 593, 537, 500, 486

**MS (CI):** *m/z* (rel. int. in %) = 403 [M^+^ + H] (100), 296 (100)

**MS (EI):** *m/z* (rel. int. in %) = 402 [M^+^**^•^**] (30), 374 (10), 295 (100), 283 (15), 167 (25), 106 (15), 91 (35)

**HR-MS (EI):** *m/z* = 402.0604 (calculated for C_19_H_19_IN_2_: 402.0593)

**HPLC purity:** > 99 %

**(±)-6-Bromo-*N^1^*-cyclohexyl-2,3,4,9-tetrahydro-1*H*-carbazol-1-amine (gea_87)**

C_18_H_23_BrN_2_; M*_r_* = 347.29 g/mol

A suspension of 417 mg (1.58 mmol) 6-bromo-2,3,4,9-tetrahydro-1*H*-carbazol-1-one[^3^](#_ENREF_3), 20 mg (0.12 mmol) magnesium perchlorate, and 556 µL (4.86 mmol) cyclohexylamine in 30 mL 1,2-dichlorethane is stirred at room temperature for 72 h, then concentrated to dryness. The residue is dissolved in 30 mL methanol, treated with171 mg (4.50 mmol) sodium borohydride and stirred for 5 h. Then 20 mL of a saturated sodium bicarbonate solution are added, and the mixture is extracted with ethyl acetate (3 x 15 mL). The combined organic layers are dried over sodium sulfate, concentrated, and the residue purified by FCC (dichloromethane/ethyl acetate 1:1).

**Yield:** 320 mg (58 %), brown wax

**Melting range:** 76 – 78 °C (ref. [^4^](#_ENREF_4): no melting range given)

**^1^H-NMR (400 MHz, CDCl_3_):** δ (ppm) = 8.50 (br s, 1H, 9-NH), 7.56 (s, 1H, 5-H), 7.18 (d, *J* = 8.6 Hz, 1H, 7-H), 7.15 (d, *J* = 8.6 Hz, 1H, 8-H), 4.01 (s, 1H, 1-H), 2.82 – 2.71 (m, 1H, 1´-H), 2.70 – 2.56 (m, 2H, 4-H), 2.34 – 2.21 (m, 1H, 2-H), 2.09 – 1.95 (m, 2H, 3-H, 2´-H or 6´-H), 1.86 – 1.58 (m, 6H, 3-H, 2´-H or 6´-H, 3´-H, 4´-H, 5´-H, 1´-NH), 1.57 – 1.45 (m, 1H, 2-H), 1.40 – 1.02 (m, 5H, 2´-H, 3´-H, 4´-H, 5´-H, 6´-H).

**^13^C-NMR (100 MHz, CDCl_3_):** δ (ppm) = 138.5 (C-9a), 134.5 (C-8a), 129.6 (C-4b), 124.2 (C-7), 121.0 (C-5), 112.4 (C-6, C-8), 110.8 (C-4a), 53.5 (C-1´), 49.2 (C-1), 35.1 (C-2´ or C-6´), 33.3 (C-2´ or C-6´), 31.0 (C-2), 26.3 (C-4´), 25.3 (C-3´ or C-5´), 24.9 (C-3´ or C-5´), 21.9 (C-3), 21.0 (C-4).

**IR (CHCl_3_ film):** $\tilde{\nu}$ (cm^-1^) = 3434, 2927, 2850, 1579, 1446, 1349, 1309, 1099, 1047, 898, 793, 756

**MS (CI):** *m/z* (rel. int. in %) = 349 [M^+^ + H] (100), 347 (100), 268 (15), 250 (80), 248 (75), 128 (10), 112 (10)

**MS (EI):** *m/z* (rel. int. in %) = 348 [M^+^**^•^**] (45), 346 (45), 320 (30), 318 (30), 249 (100), 223 (10), 168 (95), 155 (10), 129 (10), 98 (15), 55 (30)

**HR-MS (EI):** *m/z* = 346.1045 (calculated for C_18_H_23_BrN_2_: 346.1045)

**HPLC purity:** > 99 %

**2,2,9-Trimethyl-2,3,4,9-tetrahydro-1*H*-carbazol-1-one (gea_88)**

C_15_H_17_NO; M*_r_* = 227.30 g/mol

Sodium hydride (400 mg; 60% in mineral oil; 10.0 mmol) is added to a stirred solution of 370 mg (2.00 mmol) 2,3,4,9-tetrahydro-1*H*-carbazol-1-one [^1^](#_ENREF_1) in 10 mL anhydrous THF under nitrogen, and after 30 min 0.63 µL (10 mmol) iodomethane are added, and the mixture is stirred for another 15 h. Then 20 mL water is added, and the mixture is extracted with ethyl acetate (3 x 20 mL). The combined organic layers are dried over sodium sulfate, concentrated, and the residue purified by FCC (dichloromethane).

**Yield:** 440 mg (97 %), dark brown wax

**^1^H-NMR(500 MHz, CDCl_3_):** δ (ppm) = 7.63 (d, *J* = 8.0 Hz, 1H, 5-H), 7.38 (t, *J* = 7.7 Hz, 1H, 7-H), 7.33 (d, *J* = 8.4 Hz, 1H, 8-H), 7.13 (t, *J* = 7.5 Hz, 1H, 6-H), 4.05 (s, 3H, N-CH_3_), 3.02 (t, *J* = 6.2 Hz, 2H, 4-H), 2.07 (t, *J* = 6.2 Hz, 1H, 3-H), 1.25 (s, 6H, 2 x 2-CH_3_).

**^13^C-NMR (100 MHz, CDCl_3_):** δ (ppm) = 197.7 (C=O), 140.0 (C-8a), 129.0 (C-9a), 127.4 (C-4a), 126.3 (C-7), 124.5 (C-4b), 121.1 (C-5), 119.9 (C-6), 110.2 (C-8), 43.3 (C-2), 38.2 (C-3), 31.5 (N-CH_3_), 24.5 (2 x 2-CH_3_), 18.6 (C-4).

**IR (KBr):** $\tilde{\nu}$ (cm^-1^) = 3056, 2960, 2923, 2848, 1655, 1613, 1536, 1470, 1451, 1427, 1386, 1373, 1352, 1312, 1282, 1249, 1226, 1215, 1130, 1043, 1001, 972, 930, 854, 740

**MS (CI):** *m/z* (rel. int. in %) = 228 [M^+^ + H] (100)

**MS (EI):** *m/z* (rel. int. in %) = 227 [M^+^**^•^**] (80), 212 (15), 184 (50), 171 (15), 143 (100), 128 (20), 115 (20), 102 (10), 77 (10)

**HR-MS (EI):** *m/z* = 227.1310 (calculated for C_15_H_17_NO: 227.1310)

**HPLC purity:**  > 99 %

**6-Bromo-9-methyl-2,3,4,9-tetrahydro-1H-carbazol-1-one (gea_89)**

C_13_H_12_BrNO; M*_r_* = 278.14 g/mol

A solution of 396 mg (1.50 mmol) 6-bromo-2,3,4,9-tetrahydro-1*H*-carbazol-1-one [^3^](#_ENREF_3) in 10 mL anhydrous acetone is cooled to 0 °C, and 450 mg (11.3 mmol) sodium hydride are added with stirring. After warming to room temperature over 30 min 469 µL (7.53 mmol) iodomethane are added, and the mixture is stirred for 4 h. Then 20 mL water are added, and the mixture is extracted with ethyl acetate (3 x 20 mL). The combined organic layers are dried over sodium sulfate, concentrated, and the residue purified by FCC (dichloromethane/ethyl acetate 3:1). The pure fraction is crystallized from ethyl acetate.

**Yield:** 260 mg (63 %), pale brown solid

**Melting range:** 127 – 128 °C (ref. [^6^](#_ENREF_6): 121 – 123 °C)

**^1^H-NMR (500 MHz, CDCl_3_):** δ (ppm) = 7.78 (dd, *J* = 1.9 Hz, 0.5 Hz, 1H, 5-H), 7.45 (dd, *J* = 8.9 Hz, 1.9 Hz, 1H, 7-H), 7.22 (dd, *J* = 8.9 Hz, 0.5 Hz, 1H, 8-H), 4.04 (s, 3H, N-CH_3_), 2.96 (t, *J* = 6.1 Hz, 2H, 4-H), 2.65 (t, *J* = 6.5 Hz, 2H, 2-H), 2.25 – 2.17 (m, *J* = 6.3, 2H, 3-H).

**^13^C-NMR (100 MHz, CDCl_3_):** δ (ppm) = 192.3 (C=O), 138.1 (C-8a), 131.0 (C-9a), 129.3 (C-7), 128.1 (C-4a), 126.2 (C-4b), 123.7 (C-5), 113.1 (C-6), 111.8 (C-8), 39.9 (C-2), 31.7 (N-CH_3_), 24.6 (C-3), 21.6 (C-4).

**IR (KBr):** $\tilde{\nu}$ (cm^-1^) = 3425, 3078, 2930, 1735, 1655, 1531, 1474, 1432, 1405, 1374, 1333, 1313, 1271, 1255, 1229, 1186, 1115, 1081, 1041, 1003, 941, 893, 862, 836, 795, 711, 664, 641, 578, 543, 465

**MS (CI):** *m/z* (rel. int. in %) = 280 [M^+^ + H] (100), 278 (100), 199 (15)

**MS (EI):** *m/z* (rel. int. in %) = 279 [M^+^**^•^**] (100), 277 (95), 250 (20), 248 (20), 237 (20), 235 (20), 223 (30), 221 (30), 168 (15), 142 (15), 127 (20), 115 (15), 101 (10), 75 (10)

**HR-MS (EI):** *m/z* = 277.0097 (calculated for C_13_H_12_BrNO: 277.0102)

**HPLC purity:** > 99 %

**(±)-*N^1^*-Benzyl-6-bromo-9-methyl-2,3,4,9-tetrahydro-1*H*-carbazol-1-amine (gea_90)**

C_20_H_21_BrN_2_; M*_r_* = 369.30 g/mol

A suspension of 209 mg (0.751 mmol) 6-bromo-9-methyl-2,3,4,9-tetrahydro-1*H*-carbazol-1-one **(gea_89)**, 50 mg (0.23 mmol) magnesium perchlorate, and 260 mg (2.43 mmol) benzylamine in 20 mL 1,2-dichlorethane is stirred at room temperature for 72 h, then concentrated to dryness. The residue is dissolved in 20 mL methanol, treated with 60 mg (1.6 mmol) sodium borohydride and stirred for 10 h. Then 20 mL of a saturated sodium bicarbonate solution are added, and the mixture is extracted with ethyl acetate (3 x 15 mL). The combined organic layers are dried over sodium sulfate, concentrated, and the residue purified by FCC (isohexane/ethyl acetate/EDMA 3:1:0.5).

**Yield:** 140 mg (48 %), pale brown solid

**Melting range:** 91 – 93 °C

**^1^H-NMR (500 MHz, CDCl_3_):** δ (ppm) = 7.63 (d, *J* = 1.9 Hz, 1H, 5-H), 7.48 – 7.17 (m, 6H, 7-H, 2´´-H, 3´´-H, 4´´-H, 5´´-H, 6´´-H), 7.15 (d, *J* = 8.7 Hz, 1H, 8-H), 4.92 (t, *J* = 3.7 Hz, 1H, 1-H), 4.03 (d, *J* = 13.0 Hz, 1H, 1´-H), 3.74 (s, 3H, 9-CH_3_), 3.30 (d, *J* = 13.0 Hz, 1H, 1´-H), 2.81 – 2.73 (m, 1H, 4-H), 2.60 – 2.50 (m, 1H, 4-H), 2.10 – 2.00 (m, 2H, 2-H), 1.92 – 1.88 (m, 3H, 3-H, NH).

**^13^C-NMR (100 MHz, CDCl_3_):** δ (ppm) = 139.3 (C-1´), 136.9 (C-9a), 136.2 (C-8a), 129.0 (C-2´´, C-6´´), 128.4 (C-3´´, C-5´´), 128.1 (C-4b), 127.1 (C-4´´), 125.0 (C-7), 121.7 (C-5), 112.3 (C-6), 111.7 (C-4a), 110.7 (C-8), 62.0 (C-1), 57.9 (C-1´), 33.4 (C-2), 29.8 (9-CH_3_), 21.2 (C-4), 18.6 (C-3).

**IR (KBr):** $\tilde{\nu}$ (cm^-1^) = 3375, 3028, 2929, 2836, 1469, 1452, 1411, 1369, 1267, 1242, 1192, 1169, 1068, 1051, 995, 978, 926, 850, 833, 804, 793, 737, 698, 661, 580

**MS (CI):** *m/z* (rel. int. in %) = 296 [M^+^ + H – Phe] (20), 294 (20), 262 (100), 209 (10), 181 (40), 150 (10), 127 (10)

**MS (EI):** *m/z* (rel. int. in %) = 295 [M^+^**^•^** – Phe] (15), 293 (15), 261 (100), 237 (30), 180 (20), 148 (25), 115 (10), 91 (85), 75 (20), 57 (20)

**HR-MS (EI):** *m/z* = 368.0880 (calculated for C_20_H_21_BrN_2_: 368.0888)

**HPLC purity:** > 99 %

**(±)-*N^1^*-Benzyl-6-bromo-*N^1^*-methyl-2,3,4,9-tetrahydro-1*H*-carbazol-1-amine (gea_92)**

C_20_H_21_BrN_2_; M*_r_* = 369.30 g/mol

To a solution of 353 mg (0.901 mmol) *N*-benzyl-6-bromo-2,3,4,9-tetrahydro-1*H*-carbazol-1-amine hydrochloride **(gea_85)** in 10 mL anhydrous THF under nitrogen are added 500 µL (2.90 mmol) *N*-ethyl-*N*,*N*-diisopropylamine, and then 57 µL (0.91 mmol) iodomethane, the mixture is stirred at room temperature for 20 h, and then concentrated. The residue is taken up in a mixture of 15 mL ethyl acetate, 20 mL water and 20 mL brine, and extracted with ethyl acetate (3 x 15 mL). The combined organic layers are dried over sodium sulfate, concentrated, and the residue purified by FCC (isohexane/ethyl acetate/EDMA 12:1:0.5).

**Yield:** 140 mg (42 %), pale yellow wax

**Melting range:** 110 – 112 °C

**^1^H-NMR (500 MHz, CDCl_3_):** δ (ppm) = 8.39 (br s, 1H, NH), 7.57 (s, 1H, 5-H), 7.39 – 7.32 (m, 4H, 2´´-H, 3´´-H, 5´´-H, 6´´-H), 7.28 – 7.24 (m, 1H, 4´´-H), 7.21 – 7.18 (m, 2H, 7-H, 8-H), 4.14 (s, 1H, 1-H), 3.67 (d, *J* = 13.3 Hz, 1H, 1´-H), 3.59 (d, *J* = 13.3 Hz, 1H, 1´-H), 2.71 – 2.55 (m, 2H, 4-H), 2.19 (s, 3H, N-CH_3_), 2.16 – 2.05 (m, 2H, 2-H), 1.85 – 1.66 (m, 2H, 3-H).

**^13^C-NMR (100 MHz, CDCl_3_):** δ (ppm) = 139.7 (C-1´´), 137.2 (C-9a), 134.5 (C-8a), 129.8 (C-4b), 128.8 (C-2´´, C-6´´), 128.6 (C-3´´, C-5´´), 127.3 (C-4´´), 124.2 (C-7), 121.0 (C-5), 112.4 (C-4a), 112.3 (C-6, C-8), 58.4 (C-1), 57.9 (C-1´), 37.5 (N-CH_3_), 22.9 (C-2), 21.0 (C-4), 20.6 (C-3).

**IR (CHCl_3_ film):** $\tilde{\nu}$ (cm^-1^) = 3444, 3027, 2933, 2844, 2794, 2360, 2341, 1492, 1468, 1444, 1369, 1350, 1307, 1269, 1153, 1124, 1049, 1025, 793, 742, 698

**MS (CI):** *m/z* (rel. int. in %) = 371 [M^+^ + H] (100), 369 (100), 290 (10), 249 (80), 122 (35)

**MS (EI):** *m/z* (rel. int. in %) = 370 [M^+^**^•^**] (30), 368 (30), 250 (100), 248 (100), 168 (50), 120 (15), 91 (30), 65 (10)

**HR-MS (EI):** *m/z* = 368.0878 (calculated for C_20_H_21_BrN_2_: 368.0888)

**HPLC purity:** > 99 %

**(±)-*N^1^*-(1-Benzylpiperidin-4-yl)-6-iodo-2,3,4,9-tetrahydro-1*H*-carbazol-1-amine (gea_96)**

C_24_H_28_IN_3_; M*_r_* = 485.40 g/mol

A suspension of 246 mg (0.791 mmol) 6-iodo-2,3,4,9-tetrahydro-1*H*-carbazol-1-one [^5^](#_ENREF_5), 80 mg (0.36 mmol) magnesium perchlorate, and 392 µL (1.92 mmol) 4-amino-1-benzylpiperidine in 20 mL 1,2-dichlorethane is stirred at room temperature for 48 h, then concentrated to dryness. The residue is dissolved in 15 mL methanol, treated with 84 mg (2.2 mmol) sodium borohydride and stirred for 5 h. Then 20 mL of a saturated sodium bicarbonate solution are added, and the mixture is extracted with ethyl acetate (3 x 15 mL). The combined organic layers are dried over sodium sulfate, concentrated, and the residue purified by FCC (dichloromethane/ethyl acetate/EDMA 9:1:0.5).

**Yield:** 145 mg (38 %), brown solid

**Melting range:** 55 – 57 °C

**^1^H-NMR (500 MHz, CDCl_3_):** δ (ppm) = 8.41 (br s, 1H, 9-NH), 7.77 (d, *J* = 1.6 Hz, 1H, 5-H), 7.35 (dd, *J* = 8.5 Hz, 1.6 Hz, 1H, 7-H), 7.33 – 7.30 (m, 4H, 2´´´-H, 3´´´-H, 5´´´-H, 6´´´-H), 7.27 – 7.23 (m, 1H, 4´´´-H), 7.08 (d, *J* = 8.5 Hz, 1H, 8-H), 3.98 (t, *J* = 6.7 Hz, 1H, 1-H), 3.52 (s, 2H, 1´´-H), 2.91 – 2.82 (m, 2H, 2´-H, 6´-H), 2.81 – 2.75 (m, 1H, 4´-H), 2.66 – 2.58 (m, 2H, 4-H), 2.31 – 2.24 (m, 1H, 2-H), 2.10 – 1.99 (m, 4H, 1-NH, 3-H, 2´-H, 6´-H), 1.96 – 1.90 (m, 1H, 3´-H or 5´-H), 1.79 – 1.72 (m, 1H, 3-H), 1.70 – 1.65 (m, 1H, 3´-H or 5´-H), 1.57 – 1.45 (m, 3H, 2-H, 3´-H, 5´-H).

**^13^C-NMR (125 MHz, CDCl_3_):** δ (ppm) = 138.4 (C-1´´´), 137.8 (C-9a), 134.7 (C-8a), 130.3 (C-4b), 129.5 (C-7), 129.1 (C-2´´´, C-6´´´), 128.2 (C-3´´´´, C-5´´´), 127.2 (C-5), 127.0 (C-4´´´), 112.7 (C-8), 110.2 (C-4a) 82.3 (C-6), 63.1 (C-1´´), 52.2 (C-2´ or C-6´), 52.0 (C-2´ or C-6´), 51.6 (C-4´), 49.2 (C-1), 34.0 (C-3´ or C-5´), 32.4 (C-3´ or C-5´), 31.0 (C-2), 21.8 (C-3), 20.7 (C-4).

**IR (KBr):** $\tilde{\nu}$ (cm^-1^) = 3424, 3025, 2924, 2843, 2801, 1628, 1576, 1493, 1452, 1439, 1366, 1341, 1307, 1268, 1236, 1151, 1095, 1073, 1027, 977, 887, 861, 790, 740, 697, 626, 582

**MS (CI):** *m/z* (rel. int. in %) = 486 [M^+^ + H] (55), 296 (100), 217 (10), 191 (100), 175 (85), 146 (10)

**MS (EI):** *m/z* (rel. int. in %) = 485 [M^+^**^•^**] (2), 311 (40), 296 (15), 190 (20), 175 (85), 146 (50), 91 (100), 70 (15), 56 (10)

**HR-MS (EI):** *m/z* = 485.1347 (calculated for C_24_H_28_IN_3_: 485.1328)

**HPLC purity:** > 99 %

**(±)-*N^1^*-(1-Benzylpiperidin-4-yl)-6-bromo-2,3,4,9-tetrahydro-1*H*-carbazol-1-amine (gea_97)**

C_24_H_28_BrN_3_; M*_r_* = 438.40 g/mol

A suspension of 209 mg (0.791 mmol) 6-bromo-2,3,4,9-tetrahydro-1*H*-carbazol-1-one[^3^](#_ENREF_3), 80 mg (0.36 mmol) magnesium perchlorate, and 392 µL (1.92 mmol) 4-amino-1-benzylpiperidine in 20 mL 1,2-dichlorethane is stirred at room temperature for 48 h, then concentrated to dryness. The residue is dissolved in 15 mL methanol, treated with 84 mg (2.2 mmol) sodium borohydride and stirred for 5 h. Then 20 mL of a saturated sodium bicarbonate solution are added, and the mixture is extracted with ethyl acetate (3 x 15 mL). The combined organic layers are dried over sodium sulfate, concentrated, and the residue purified by FCC (dichloromethane/ethyl acetate/EDMA 9:1:0.5).

**Yield:** 87 mg (25 %), brown solid

**Melting range:** 135 – 137 °C

**^1^H-NMR (400 MHz, CDCl_3_):** δ (ppm) = 8.48 (br s, 1H, 9-NH), 7.56 (s, 1H, 5-H), 7.34 – 7.31 (m, 4H, 2´´´-H, 3´´´-H, 5´´´-H, 6´´´-H), 7.31 – 7.27 (m, 1H, 4´´´-H), 7.21 – 7.15 (m, 2H, 7-H, 8-H), 4.00 (t, *J* = 6.6 Hz, 1H, 1-H), 3.52 (s, 2H, 1´´-H), 2.92 – 2.74 (m, 3H, 2´-H, 4´-H, 6´-H), 2.68 – 2.57 (m, 2H, 4-H), 2.32 – 2.23 (m, 1H, 2-H), 2.13 – 2.00 (m, 4H, 1-NH, 3-H, 2´-H, 6´-H), 1.99 – 1.91 (m, 1H, 3´-H or 5´-H), 1.79 – 1.64 (m, 2H, 3-H, 3´-H or 5´-H), 1.55 – 1.45 (m, 3H, 2-H, 3´-H, 5´-H).

**^13^C-NMR (100 MHz, CDCl_3_):** δ (ppm) = 138.4 (C-1´´´), 138.0 (C-9a), 134.3 (C-8a), 129.4 (C-4b), 129.1 (C-2´´´, C-6´´´), 128.2 (C-3´´´, C-5´´´), 127.0 (C-4´´´), 124.0 (C-7), 120.8 (C-5), 112.2 (C-8, C-6), 110.6 (C-4a), 63.0 (C-1´´), 52.2 (C-2´ or C-6´), 52.0 (C-2´ or C-6´), 51.6 (C-4´), 49.2 (C-1), 33.9 (C-3´ or C-5´), 32.3 (C-3´ or C-5´), 30.9 (C-2), 21.8 (C-3), 20.7 (C-4).

**IR (CHCl_3_ film):** $\tilde{\nu}$ (cm^-1^) = 3424, 3283, 3027, 2933, 2848, 2804, 2762, 1659, 1629, 1493, 1466, 1451, 1367, 1342, 1310, 1152, 1096, 1072, 1047, 1028, 978, 892, 858, 791, 741, 698

**MS (CI):** *m/z* (rel. int. in %) = 440 [M^+^ + H] (70), 438 (40), 358 (10), 265 (10), 249 (90), 203 (10), 191 (100), 175 (95), 102 (15)

**MS (EI):** *m/z* (rel. int. in %) = 439 [M^+^**^•^**] (2), 437(1), 265 (20), 263 (20), 249 (10), 189 (20), 175 (65), 146 (50), 120 (10), 91 (100), 83 (20), 70 (10), 56 (10)

**HR-MS (EI):** *m/z* = 437.1474 (calculated for C_24_H_28_BrN_3_: 437.1467)

**HPLC purity:** > 99 %

**(±)-6-Bromo-2,3,4,9-tetrahydro-1*H*-carbazol-1-amine hydrochloride (gea_99)**

C_12_H_14_BrClN_2_; M*_r_* = 301.61 g/mol

(free amine: C_12_H_13_BrN_2_; M*_r_* = 265.15 g/mol)

A solution of 500 mg (1.89 mmol) 6-bromo-2,3,4,9-tetrahydro-1*H*-carbazol-1-one[^3^](#_ENREF_3), 1.46 g (18.9 mmol) ammonium acetate, and 597 mg (9.50 mmol) sodium cyanoborohydride in 40 mL methanol is refluxed for 15 h, then diluted with 25 mL water, and extracted with ethyl acetate (3 x 20 mL). The combined organic layers are dried over sodium sulfate, concentrated, and the residue taken up in a small amount of methanol. Conc. hydrochloric acid is added dropwise to this solution until precipitation ceases. The precipitate is collected by filtration, washed with a small portion of methanol, and dried.

**Yield:** 265 mg (46 %), brown powder

**Melting range:** 210 – 212 °C (ref. [^7^](#_ENREF_7): no melting range given)

**^1^H-NMR (500 MHz, DMSO-d_6_):** δ (ppm) = 11.30 (br s, 1H, 9-NH), 8.68 (br s, 3H, 1-NH_3_), 7.66 (d, *J* = 1.9 Hz, 1H, 5-H), 7.38 (d, *J* = 8.6 Hz, 1H, 8-H), 7.24 (dd, *J* = 8.6 Hz, 1.9 Hz, 1H, 7-H), 4.55 (s, 1H, 1-H), 2.71 – 2.56 (m, 2H, 4-H), 2.22 – 2.12 (m, 1H, 2-H), 2.06 – 1.97 (m, 1H, 3-H), 1.96 – 1.88 (m, 1H, 2-H), 1.84 – 1.75 (m, 1H, 3-H).

**^13^C-NMR (100 MHz, DMSO-d_6_):** δ (ppm) = 134.3 (C-8a), 131.1 (C-9a), 127.8 (C-4b), 124.5 (C-7), 120.8 (C-5), 113.4 (C-8), 112.2 (C-4a), 111.3 (C-6), 43.8 (C-1), 27.6 (C-2), 19.9 (C-4), 19.3 (C-3).

**IR (KBr):** $\tilde{\nu}$ (cm^-1^) = 3318, 2924, 2008, 1605, 1497, 1447, 1382, 1355, 1313, 1274, 1197, 1053, 989, 876, 801, 793, 742, 657, 583, 532, 511

**MS (CI):** *m/z* (rel. int. in %) = 266 [M^+^ + H] (100), 250 (50), 248 (65), 198 (10), 186 (40), 102 (10)

**MS (EI):** *m/z* (rel. int. in %) = 266 [M^+^**^•^**] (25), 264 (25), 249 (60), 247 (60), 237 (15), 168 (100), 129 (30), 115 (15), 103 (20), 91 (25), 72 (85), 63 (20), 58 (50), 51 (25)

**HR-MS (EI):** *m/z* = 264.0262 (calculated for C_12_H_14_BrClN_2_: 264.0262)

**HPLC purity:** > 99 %

**(±)-6-Bromo-*N^1^*-(1-phenethylpiperidin-4-yl)-2,3,4,9-tetrahydro-1*H*-carbazol-1-amine (gea_101)**

C_25_H_30_BrN_3_; M*_r_* = 452.43 g/mol

A suspension of 220 mg (0.729 mmol) 6-bromo-2,3,4,9-tetrahydro-1*H*-carbazol-1-amine hydrochloride **(gea_99)**, 80 mg (0.36 mmol) magnesium perchlorate, and 360 mg (1.77 mmol) 1-phenethyl-4-piperidone in 10 mL of a mixture of 1,2-dichlorethane and methanol (2:1) is stirred at room temperature for 48 h, then 84 mg (2.2 mmol) sodium borohydride are added, and the mixture stirred for 5 h. Then 20 mL of a saturated sodium bicarbonate solution are added, and the mixture is extracted with ethyl acetate (3 x 15 mL). The combined organic layers are dried over sodium sulfate, concentrated, and the residue subjected to FCC (dichloromethane/ethyl acetate/EDMA 9:1:0.5). Final purification is performed by another FCC (cyclohexane/ethyl acetate/triethylamine/EDMA 1:2:0.3:0.3).

**Yield:** 50 mg (15%), brown wax

**^1^H-NMR (400 MHz, CDCl_3_):** δ (ppm) = 8.56 (br s, 1H, 9-NH), 7.57 (s, 1H, 5-H), 7.32 – 7.26 (m, 3H, 3´´´-H, 4´´´-H, 5´´´-H), 7.23 – 7.19 (m, 4H, 7-H, 8-H, 2´´´-H, 6´´´-H), 4.02 (t, *J* = 6.6 Hz, 1H, 1-H), 3.02 – 2.97 (m, 2H, 2´-H, 6´-H), 2.81 – 2.77 (m, 3H, 4´-H, 2´´-H), 2.67 – 2.57 (m, 4H, 4-H, 1´´-H), 2.34 – 2.24 (m, 1H, 2-H), 2.17 – 2.05 (m, 4H, 1-NH, 3-H, 2´-H, 6´-H), 2.04 – 2.00 (m, 1H, 3´-H or 5´-H), 1.80 – 1.71 (m, 2H, 3-H, 3´-H or 5´-H), 1.58 – 1.47 (m, 3H, 2-H, 3´-H, 5´-H).

**^13^C-NMR (100 MHz, CDCl_3_):** δ (ppm) = 140.5 (C-1´´´), 138.3 (C-9a), 134.3 (C-8a), 129.4 (C-4b), 128.7 (C-2´´´, C-6´´´), 128.4 (C-3´´´, C-5´´´), 126.0 (C-4´´´), 124.0 (C-7), 120.8 (C-5), 112.2 (C-8), 112.1 (C-6), 110.5 (C-4a), 60.7 (C-1´´), 52.4 (C-2´ or C-6´), 52.2 (C-2´ or C-6´), 51.5 (C-4´), 49.3 (C-1), 33.9 (C-3´ or C-5´), 33.9 (C-2´´), 32.5 (C-3´ or C-5´), 31.0 (C-2), 21.8 (C-3), 20.8 (C-4).

**IR (CHCl_3_ film):** $\tilde{\nu}$ (cm^-1^) = 3282, 3026, 2929, 2852, 2807, 1639, 1495, 1466, 1452, 1373, 1309, 1246, 1105, 1072, 1046, 894, 858, 792, 746, 698

**MS (CI):** *m/z* (rel. int. in %) = 454 [M^+^ + H] (50), 250 (100), 189 (20), 169 (20)

**MS (EI):** *m/z* (rel. int. in %) = 451 [M^+^**^•^**] (1), 264 (10), 250 (50), 248 (65), 189 (45), 168 (35), 113 (65), 98 (30), 85 (25), 70 (100), 56 (20)

**HR-MS (EI):** *m/z* = 451.1612 (calculated for C_25_H_30_BrN_3_: 451.1623)

**HPLC purity:** > 99 %

**(±)-6-Iodo-*N^1^*-(1-phenethylpiperidin-4-yl)-2,3,4,9-tetrahydro-1*H*-carbazol-1-amine (gea_102)**

C_25_H_30_IN_3_; M*_r_* = 499.43 g/mol

A solution of 200 mg (0.574 mmol) 6-iodo-2,3,4,9-tetrahydro-1*H*-carbazol-1-amine hydrochloride **(gea_100)**, 80 mg (0.36 mmol) magnesium perchlorate, and 281 mg (1.38 mmol) 1-phenethyl-4-piperidone in 10 mL of a mixture of 1,2-dichloroethane and methanol (2:1) is stirred at room temperature for 48 h, then 84 mg (2.2 mmol) sodium borohydride are added, and the mixture stirred for 12 h. Then 20 mL of a saturated sodium bicarbonate solution are added, and the mixture is extracted with ethyl acetate (3 x 15 mL). The combined organic layers are dried over sodium sulfate, concentrated, and the residue subjected to FCC (dichloromethane/ethyl acetate/EDMA 9:1:0.5). Final purification is performed by another FCC (cyclohexane/ethyl acetate/triethylamine/EDMA 1:2:0.3:0.3).

**Yield:** 45 mg (16 %), dark red wax

**^1^H-NMR (500 MHz, CDCl_3_):** δ (ppm) = 8.39 (br s, 1H, 9-NH), 7.78 (d, *J* = 1.7 Hz, 1H, 5-H), 7.37 (dd, *J* = 8.4 Hz, 1.7 Hz, 1H, 7-H), 7.29 – 7.27 (m, 2H, 3´´´-H, 5´´´-H), 7.23 – 7.21 (m, 3H, 2´´´-H, 4´´´-H, 6´´´-H), 7.10 (d, *J* = 8.4 Hz, 1H, 8-H), 4.01 (t, *J* = 6.7 Hz, 1H, 1-H), 3.03 – 2.96 (m, 2H, 2´-H, 6´-H), 2.88 – 2.82 (m, 3H, 4´-H, 2´´-H), 2.67 – 2.60 (m, 4H, 4-H, 1´´-H), 2.33 – 2.28 (m, 1H, 2-H), 2.25 – 2.11 (m, 4H, 1-NH, 3-H, 2´-H, 6´-H), 2.04 – 2.00 (m, 1H, 3´-H or 5´-H), 1.77 – 1.73 (m, 2H, 3-H, 3´-H or 5´-H), 1.62 – 1.52 (m, 3H, 2-H, 3´-H, 5´-H).

**^13^C-NMR (100 MHz, CDCl_3_):** δ (ppm) = 140.4 (C-1´´´), 137.8 (C-9a), 134.7 (C-8a), 130.3 (C-4b), 129.5 (C-7), 128.7 (C-2´´´, C-6´´´), 128.4 (C-3´´´, C-5´´´), 127.2 (C-5), 126.0 (C-4´´´), 112.8 (C-8), 110.3 (C-4a), 82.3 (C-6), 60.7 (C-1´´), 52.4 (C-2´ or C-6´), 52.2 (C-2´ or C-6´), 51.5 (C-4´), 49.2 (C-1), 33.9 (C-3´ or C-5´), 33.9 (C-2´´), 32.5 (C-3´ or C-5´), 31.0 (C-2), 21.9 (C-3), 20.7 (C-4).

**IR (CHCl_3_ film):** $\tilde{\nu}$ (cm^-1^) = 3301, 3060, 3026, 2928, 2852, 2813, 2361, 2342, 1658, 1603, 1495, 1453, 1373, 1308, 1238, 1109, 1071, 797, 748, 699

**MS (CI):** *m/z* (rel. int. in %) = 500 [M^+^ + H] (65), 296 (85), 223 (30), 205 (100), 189 (50), 170 (70), 153 (60), 127 (35), 113 (60), 102 (30)

**MS (ESI):** *m/z* (rel. int. in %) = 500 [M^+^ + H], 433, 409, 296, 206, 188, 118, 105, 102

**HR-MS (EI):** *m/z* = 499.1506 (calculated for C_25_H_30_IN_3_: 499.1485)

**(±)-*N^1^*-(1-Benzylpiperidin-4-yl)-6-chloro-2,3,4,9-tetrahydro-1*H*-carbazol-1-amine (gea_129)**

C_24_H_28_ClN_3_; M*_r_* = 393.95 g/mol

A solution of 220 mg (1.00 mmol) 6-chloro-2,3,4,9-tetrahydro-1*H*-carbazol-1-one [^8^](#_ENREF_8), 11 mg (0.050 mmol) magnesium perchlorate, and 490 µL (2.40 mmol) 4-amino-1-benzylpiperidine in 12 mL of a mixture of 1,2-dichloroethane and methanol (2:1) is stirred at room temperature for 48 h, then 72 mg (1.9 mmol) sodium borohydride are added, and the mixture stirred for 12 h. Then 20 mL of a saturated sodium bicarbonate solution are added, and the mixture is extracted with ethyl acetate (3 x 15 mL). The combined organic layers are dried over sodium sulfate, concentrated, and the residue subjected to FCC (dichloromethane/ethyl acetate/EDMA 9:1:0.5).

**Yield:** 45 mg (11 %), brown solid

**Melting range:** 46 – 48 °C

**^1^H-NMR (400 MHz, CD_2_Cl_2_):** δ (ppm) = 8.53 (s, 1H, 9-NH), 7.39 (d, *J* = 2.0 Hz, 1H, 5-H), 7.32 – 7.29 (m, 4H, 2´´´-H, 3´´´-H, 5´´´-H, 6´´´-H), 7.26 – 7.20 (m, 2H, 8-H, 4´´´-H), 7.03 (dd, *J* = 8.6 Hz, 2.0 Hz, 1H, 7-H), 4.00 (t, *J* = 6.8 Hz, 1H, 1-H), 3.49 (s, 2H, 1´´-H), 2.87 – 2.76 (m, 3H, 2´-H, 4´-H, 6´-H), 2.66 – 2.60 (m, 2H, 4-H), 2.33 – 2.24 (m, 1H, 2-H), 2.12 – 1.98 (m, 4H, 1-NH, 3-H, 2´-H, 6´-H), 1.98 – 1.92 (m, 1H, 3´-H or 5´-H), 1.76 – 1.66 (m, 2H, 3-H, 3´-H or 5´-H), 1.53 – 1.41 (m, 3H, 2-H, 3´-H, 5´-H).

**^13^C-NMR (125 MHz, CD_2_Cl_2_):** δ (ppm) = 139.6 (C-1´´´), 139.5 (C-9a), 134.6 (C-8a), 129.6 (C-2´´´, C-6´´´), 129.4 (C-4b), 128.7 (C-3´´´, C-5´´´), 127.4 (C-4´´´), 124.8 (C-6), 121.6 (C-7), 118.1 (C-5), 112.3 (C-8), 111.0 (C-4a), 63.5 (C-1´´), 52.8 (C-2´ or C-6´), 52.6 (C-2´ or C-6´), 52.2 (C-4´), 49.9 (C-1), 34.7 (C-3´ or C-5´), 33.1 (C-3´ or C-5´), 31.6 (C-2), 22.4 (C-3), 21.3 (C-4).

**IR (CHCl_3_ film):** $\tilde{\nu}$ (cm^-1^) = 3287, 3061, 3028, 2927, 2850, 2804, 2360, 2341, 1669, 1577, 1493, 1452, 1367, 1311, 1095, 1065, 1028, 793, 741, 698

**MS (CI):** *m/z* (rel. int. in %) = 396 [M^+^ + H] (20), 394 (40), 218 (30), 205 (100), 191 (70), 175 (50), 156 (20), 146 (10)

**MS (EI):** *m/z* (rel. int. in %) = 394 [M^+^**^•^**] (2), 219 (30), 204 (10), 189 (15), 175 (60), 146 (45), 91 (100), 84 (15), 70 (10), 55 (10)

**HR-MS (EI):** *m/z* = 393.1973 (calculated for C_24_H_28_ClN_3_: 393.1972)

**HPLC purity:** > 99 %

**(±)-*N^1^*-(1-Benzylpiperidin-4-yl)-6-(trifluoromethyl)-2,3,4,9-tetrahydro-1*H*-carbazol-1-amine (gea_130)**

C_25_H_28_F_3_N_3_; M*_r_* = 427.51 g/mol

A solution of 253 mg (1.00 mmol) 6-(trifluoromethyl)-2,3,4,9-tetrahydro-1*H*-carbazol-1-one [^7^](#_ENREF_7), 11 mg (0.050 mmol) magnesium perchlorate, and 490 µL (2.40 mmol) 4-amino-1-benzylpiperidine in 12 mL of a mixture of 1,2-dichloroethane and methanol (2:1) is stirred at room temperature for 48 h, then 72 mg (1.9 mmol) sodium borohydride are added, and the mixture stirred for 12 h. Then 20 mL of a saturated sodium bicarbonate solution are added, and the mixture is extracted with ethyl acetate (3 x 15 mL). The combined organic layers are dried over sodium sulfate, concentrated, and the residue subjected to FCC (dichloromethane/ethyl acetate/EDMA 9:1:0.5).

**Yield:** 105 mg (25 %), green solid

**Melting range:** 46 – 48 °C

**^1^H-NMR (500 MHz, CD_2_Cl_2_):** δ (ppm) = 8.77 (br s, 1H, 9-NH), 7.74 (s, 1H, 5-H), 7.39 (d, *J* = 8.5 Hz, 1H, 8-H), 7.33 – 7.29 (m, 5H, 7-H, 2´´´-H, 3´´´-H, 5´´´-H, 6´´´-H), 7.26 – 7.22 (m, 1H, 4´´´-H), 4.04 (t, *J* = 6.8 Hz, 1H, 1-H), 3.50 (s, 2H, 1´´-H), 2.91 – 2.80 (m, 3H, 2´-H, 4´-H, 6´-H), 2.74 – 2.64 (m, 2H, 4-H), 2.35 – 2.28 (m, 1H, 2-H), 2.13 – 2.02 (m, 4H, 1-NH, 3-H, 2´-H, 6´-H), 1.99 – 1.93 (m, 1H, 3´-H or 5´-H), 1.83 – 1.75 (m, 1H, 3-H), 1.75 – 1.69 (m, 1H, 3´-H or 5´-H), 1.56 – 1.43 (m, 3H, 2-H, 3´-H, 5´-H).

**^13^C-NMR (100 MHz, CD_2_Cl_2_):** δ (ppm) = 139.5 (C-9a), 139.4 (C-1´´´), 137.5 (C-8a), 129.4 (C-2´´´, C-6´´´), 128.5 (C-3´´´, C-5´´´), 127.5 (C-4b), 127.3 (C-4´´´), 124.8 (CF_3_), 121.2 (d, ^2^*J*_CF_ = 31.4, C-6), 118.1 (d, ^3^*J*_CF_ = 3.6, C-7), 116.2 (d, ^3^*J*_CF_ = 4.3, C-5), 112.0 (C-4a), 111.3 (C-8), 63.3 (C-1´´), 52.7 (C-2´ or C-6´), 52.4 (C-2´ or C-6´), 52.1 (C-4´), 49.7 (C-1), 34.4 (C-3´ or C-5´), 32.9 (C-3´ or C-5´), 31.2 (C-2), 22.3 (C-3), 21.1 (C-4).

**IR (CHCl_3_ film):** $\tilde{\nu}$ (cm^-1^) = 3264, 3061, 3029, 2934, 2851, 2805, 2360, 2342, 1670, 1629, 1454, 1326, 1267, 1156, 1108, 1049, 807, 741, 698

**MS (CI):** *m/z* (rel. int. in %) = 428 [M^+^ + H] (35), 408 (10), 238 (50), 219 (75), 191 (100), 175 (50), 146 (10)

**MS (EI):** *m/z* (rel. int. in %) = 428 [M^+^**^•^**] (2), 253 (20), 238 (15), 189 (20), 175 (60), 146 (50), 91 (100), 83 (20), 70 (10), 55 (10)

**HR-MS (EI):** *m/z* = 427.2238 (calculated for C_25_H_28_F_3_N_3_: 427.2235)

**HPLC purity:** > 99 %

**1-Oxo-2,3,4,9-tetrahydro-1*H*-carbazole-6-carbonitrile (gea_131)**

C_13_H_10_N_2_O; M*_r_* = 210.23 g/mol

Diazonium salt solution: 10 g (85 mmol) 4-aminobenzonitrile are dissolved in 170 mL water and 22 mL conc. hydrochloric acid. This solution is cooled to 0 °C in an ice bath, treated with 8.20 g (119 mmol) sodium nitrite, and after stirring at 0 °C for 1h, with 5.7 g (95 mmol) urea, and neutralized with satd. sodium bicarbonate solution. Stirring is continued until the evolution of gas ceases.

A solution prepared by stirring a mixture of 15 mL (94 mmol) ethyl 2-oxocyclohexanecarboxylate and 6.6 g (119 mmol) potassium hydroxide in 60 mL water at 30 °C for 4h, and then cooling to 0 °C, is added to the diazonium salt solution, and the mixture is immediately adjusted to pH <3 with glacial acetic acid, and stirred at room temperature for 15 h. The precipitate is collected by filtration, dissolved in 170 mL anhydrous formic acid, and heated at 80 °C for 15 h. Formic acid is removed by vacuum distillation, and the residue dissolved in 80 mL ethyl acetate, 80 mL 2M sodium hydroxide solution and 50 mL brine are added, and the mixture is extracted with ethyl acetate (3 x 100 mL). The combined organic layers are dried over sodium sulfate, concentrated, and the residue crystallized from methanol/chloroform (1:1).

**Yield:** 3.30 g (19 %), greyish powder

**Melting range:** 257 – 260 °C (ref. [^9^](#_ENREF_9): no melting range given)

**^1^H-NMR (400 MHz, CDCl_3_):** δ (ppm) = 9.50 (s, 1H, NH), 8.06 (d, *J* = 1.4 Hz, 1H, 5-H), 7.59 (dd, *J* = 8.6 Hz, 1.4 Hz, 1H, 7-H), 7.53 (d, *J* = 8.6 Hz, 1H, 8-H), 3.04 (t, *J* = 6.1 Hz, 2H, 4-H), 2.72 (t, *J* = 6.5 Hz, 2H, 2-H), 2.37 – 2.27 (m, 2H, 3-H).

**^13^C-NMR (100 MHz, CDCl_3_):** δ (ppm) = 191.5 (C=O), 138.9 (C-8a), 132.8 (C-9a), 129.5 (C-4a), 129.0 (C-7), 127.5 (C-5), 125.8 (C-4b), 119.9 (CN), 113.6 (C-8), 103.9 (C-6), 38.2 (C-2), 24.7 (C-3), 21.2 (C-4).

**IR (KBr):** $\tilde{\nu}$ (cm^-1^) = 3254, 2954, 2217, 1644, 1617, 1572, 1545, 1478, 1438, 1329, 1261, 1177, 1137, 904, 821

**MS (CI):** *m/z* (rel. int. in %) = 211 [M^+^ + H] (100)

**MS (EI):** *m/z* (rel. int. in %) = 210 [M^+^**^•^**] (80), 181 (25), 168 (25), 154 (100), 127 (30), 77 (15)

**HR-MS (EI):** *m/z* = 210.0798 (calculated for C_13_H_10_N_2_O: 210.0793)

**HPLC purity:** 96 %

**(±)-*N^1^*-(1-Benzylpiperidin-4-yl)-6-fluoro-2,3,4,9-tetrahydro-1*H*-carbazol-1-amine (gea_132)**

C_24_H_28_FN_3_; M*_r_* = 377.50 g/mol

A solution of 203 mg (1.00 mmol) 6-fluoro-2,3,4,9-tetrahydro-1*H*-carbazol-1-one [^10^](#_ENREF_10), 11 mg (0.050 mmol) magnesium perchlorate, and 490 µL (2.40 mmol) 4-amino-1-benzylpiperidine in 12 mL of a mixture of 1,2-dichloroethane and methanol (2:1) is stirred at room temperature for 48 h, then 72 mg (1.9 mmol) sodium borohydride are added, and the mixture stirred for 12 h. Then 20 mL of a saturated sodium bicarbonate solution are added, and the mixture is extracted with ethyl acetate (3 x 15 mL). The combined organic layers are dried over sodium sulfate, concentrated, and the residue subjected to FCC (dichloromethane/ethyl acetate/EDMA 9:1:0.5). Further purification is performed by another FCC (acetone/triethylamine 1:0.1).

**Yield:** 20 mg (5 %), light-brown wax

**^1^H-NMR (500 MHz, CD_2_Cl_2_):** δ (ppm) = 8.60 (br s, 1H, 9-NH), 7.35 – 7.27 (m, 4H, 2´´´-H, 3´´´-H, 5´´´-H, 6´´´-H), 7.26 – 7.22 (m, 1H, 4´´´-H), 7.20 (dd, *J* = 8.8 Hz, 4.4 Hz, 1H, 8-H), 7.06 (dd, *J* = 9.7 Hz, 2.5 Hz, 1H, 5-H), 6.82 (td, *J* = 9.0 Hz, 2.5 Hz, 1H, 7-H), 4.01 (t, *J* = 6.8 Hz, 1H, 1-H), 3.49 (s, 2H, 1´´-H), 2.92 – 2.76 (m, 3H, 2´-H, 4´-H, 6´-H), 2.68 – 2.59 (m, 2H, 4-H), 2.30 – 2.24 (m, 1H, 2-H), 2.09 – 2.01 (m, 4H, 1-NH, 3-H, 2´-H, 6´-H), 1.96 – 1.93 (m, 1H, 3´-H or 5´-H), 1.84 – 1.73 (m, 1H, 3-H), 1.72 – 1.67 (m, 1H, 3´-H or 5´-H), 1.55 – 1.40 (m, 3H, 2-H, 3´-H, 5´-H).

**^13^C-NMR (100 MHz, CD_2_Cl_2_):** δ (ppm) = 157.9 (d, ^1^*J*_CF_ = 232.5, C-6), 139.6 (C-9a), 139.4 (C-1´´´), 132.6 (C-8a), 129.4 (C-2´´´, C-6´´´), 128.5 (C-3´´´, C-5´´´), 128.4 (d, ^3^*J*_CF_ = 9.6, C-4b), 127.2 (C-4´´´), 111.6 (d, ^3^*J*_CF_ = 9.7, C-8), 111.3 (d, ^4^*J*_CF_ = 4.6, C-4a), 109.3 (d, ^2^*J*_CF_ = 26.1, C-7), 103.3 (d, ^2^*J*_CF_ = 23.1, C-5), 63.3 (C-1´´), 52.7 (C-2´ or C-6´), 52.4 (C-2´ or C-6´), 52.0 (C-4´), 49.7 (C-1), 34.4 (C-3´ or C-5´), 32.8 (C-3´ or C-5´), 31.2 (C-2), 22.2 (C-3), 21.2 (C-4).

**IR (KBr):** $\tilde{\nu}$ (cm^-1^) = 3424, 3061, 2928, 2850, 2430, 2361, 2343, 1628, 1585, 1482, 1452, 1366, 1315, 1230, 1170, 1132, 1109, 845, 798, 740, 698, 603

**MS (CI):** *m/z* (rel. int. in %) = 378 [M^+^ + H] (50), 233 (10), 219 (10), 203 (10), 191 (60), 188 (100), 175 (30)

**MS (EI):** *m/z* (rel. int. in %) = 377 [M^+^**^•^**] (2), 203 (40), 175 (50), 146 (45), 91 (100), 82 (15), 70 (10), 55 (10)

**HR-MS (EI):** *m/z* = 377.2262 (calculated for C_24_H_28_FN_3_: 377.2267)

**(±)-1-[(1-Benzylpiperidin-4-yl)amino]-2,3,4,9-tetrahydro-1*H*-carbazole-6-carbonitrile (gea_133)**

C_25_H_28_N_4_; M*_r_* = 384.52 g/mol

A solution of 400 mg (1.90 mmol) 1-oxo-2,3,4,9-tetrahydro-1*H*-carbazol-6-carbonitrile **(gea_131)**, 11 mg (0.050 mmol) magnesium perchlorate, and 785 µL (3.80 mmol) 4-amino-1-benzylpiperidine in 15 mL of a mixture of 1,2-dichloroethane, ethanol, and ethyl acetate (1:1:1) is stirred at room temperature for 12 h, then 72 mg (1.9 mmol) sodium borohydride are added, and the mixture stirred for 12 h. Then 20 mL of a saturated sodium bicarbonate solution are added, and the mixture is extracted with ethyl acetate (3 x 15 mL). The combined organic layers are dried over sodium sulfate, concentrated, and the residue subjected to FCC (dichloromethane/ethyl acetate/EDMA 9:1:1).

**Yield:** 340 mg (47 %), brown solid

**Melting range:** 124 – 127 °C

**^1^H-NMR (500 MHz, CDCl_3_):** δ (ppm) = 8.80 (br s, 1H, 9-NH), 7.78 (s, 1H, 5-H), 7.35 – 7.31 (m, 6H, 7-H, 8-H, 2´´´-H, 3´´´-H, 5´´´-H, 6´´´-H), 7.30 – 7.26 (m, 1H, 4´´´-H), 4.02 (t, *J* = 6.6 Hz, 1H, 1-H), 3.52 (s, 2H, 1´´-H), 2.93 – 2.78 (m, 3H, 2´-H, 4´-H, 6´-H), 2.72 – 2.62 (m, 2H, 4-H), 2.35 – 2.29 (m, 1H, 2-H), 2.12 – 2.04 (m, 4H, 1-NH, 3-H, 2´-H, 6´-H), 1.99 – 1.93 (m, 1H, 3´-H or 5´-H), 1.83 – 1.75 (m, 1H, 3-H), 1.74 – 1.68 (m, 1H, 3´-H or 5´-H), 1.56 – 1.46 (m, 3H, 2-H, 3´-H, 5´-H).

**^13^C-NMR (100 MHz, CD_2_Cl_2_):** δ (ppm) = 140.0 (C-9a), 139.4 (C-1´´´), 137.8 (C-8a), 129.4 (C-2´´´, C-6´´´), 128.5 (C-3´´´, C-5´´´), 127.9 (C-4b), 127.2 (C-4´´´), 124.5 (C-7), 124.0 (C-5), 121.4 (CN), 112.0 (C-8), 111.9 (C-4a), 102.0 (C-6), 63.4 (C-1´´), 52.7 (C-2´ or C-6´), 52.3 (C-2´ or C-6´), 52.1 (C-4´), 49.4 (C-1), 34.4 (C-3´ or C-5´), 31.8 (C-3´ or C-5´), 31.0 (C-2), 22.1 (C-3), 21.0 (C-4).

**IR (CHCl_3_ film):** $\tilde{\nu}$ (cm^-1^) = 3451, 3018, 2918, 2849, 2807, 2322, 2218, 1619, 1492, 1467, 1365, 1318, 1216, 1171, 755, 699, 667

**MS (CI):** *m/z* (rel. int. in %) = 385 [M^+^ + H] (50), 195 (20), 191 (100), 175 (10)

**HR-MS (EI):** *m/z* = 384.2313 (calculated for C_25_H_28_N_4_: 384.2314)

**HPLC purity:** > 99 %

**(±)-*N^1^*-[2-(1-Benzylpiperidin-4-yl)ethyl]-6-bromo-2,3,4,9-tetrahydro-1*H*-carbazol-1-amine (gea 140)**

C_26_H_32_BrN_3_; M*_r_* = 466.46 g/mol

A solution of 317 mg (1.20 mmol) 6-bromo-2,3,4,9-tetrahydro-1*H*-carbazol-1-one [^3^](#_ENREF_3), 14 mg (0.060 mmol) magnesium perchlorate, and 500 mg (2.30 mmol) 2-(1-benzyl-4-piperidinyl)ethanamine in 10 mL of a mixture of 1,2-dichloroethane and methanol (1:1) is stirred at room temperature for 12 h, then 87 mg (2.3 mmol) sodium borohydride are added, and the mixture stirred for 12 h. Then 15 mL of a saturated sodium bicarbonate solution and 15 mL water are added, and the mixture is extracted with dichloromethane (3 x 15 mL). The combined organic layers are dried over sodium sulfate, concentrated, and the residue subjected to FCC (dichloromethane/ethyl acetate/EDMA 9:1:0.5). Further purification is performed by another FCC (dichloromethane/ethyl acetate/EDMA 1:1:0.2).

**Yield:** 250 mg (45 %), brown solid

**Melting range:** 77 – 79 °C

^1^H-NMR (400 MHz, CD_2_Cl_2_): δ (ppm) = 8.94 (br s, 1H, 9-NH), 7.55 (s, 1H, 5-H), 7.29 – 7.27 (m, 4H, 2´´´´-H, 3´´´´-H, 5´´´´-H, 6´´´´-H), 7.23 – 7.20 (m, 1H, 4´´´´-H), 7.16 – 7.14 (m, 2H, 7-H, 8-H), 3.91 (t, *J* = 6.6 Hz, 1H, 1-H), 3.43 (s, 2H, 1´´´-H), 2.84 – 2.80 (m, 3H, 1´-H, 2´´-H, 6´´-H), 2.65 – 2.57 (m, 3H, 2 x 4-H, 1´-H), 2.25 – 2.18 (m, 1H, 2-H), 2.03 – 1.99 (m, 1H, 3-H), 1.93 – 1.88 (m, 2H, 2´´-H, 6´´-H), 1.78 – 1.70 (m, 1H, 3-H), 1.65 – 1.59 (m, 3H, 2-H, 2 x 3´-H), 1.45 – 1.33 (m, 3H, 2 x 2´-H, 4´´-H), 1.25 – 1.19 (m, 2H, 3´´-H, 5´´-H).

**^13^C-NMR (100 MHz, CD_2_Cl_2_):** δ (ppm) = 139.5 (C-1´´´´), 138.4 (C-9a), 134.8 (C-8a), 129.7 (C-4b), 129.4 (C-2´´´´, C-6´´´´), 128.4 (C-3´´´´, C-5´´´´), 127.1 (C-4´´´), 124.0 (C-7), 121.1 (C-5), 112.6 (C-8), 112.1 (C-6), 111.0 (C-4a), 63.7 (C-1´´´), 54.3 (C-2´´ orr C-6´´), 54.2 (C-2´´ or C-6´´), 52.7 (C-1), 44.1 (C-1´), 37.7 (C-2´), 34.2 (C-4´´), 33.1 (C-3´´ or C-5´´), 32.7 (C-3´´ or C-5´´), 30.3 (C-2), 22.0 (C-3), 21.1 (C-4).

**IR (CHCl_3_ film):** $\tilde{\nu}$ (cm^-1^) = 3321, 3027, 2920, 2849, 2802, 2757, 2360, 2343, 1654, 1583, 1492, 1449, 1366, 1342, 1308, 1149, 1110, 1072, 1048, 1029, 792, 737, 697

**MS (CI):** *m/z* (rel. int. in %) = 468 [M^+^ + H] (15), 466 (15), 262 (10), 250 (75), 231 (30), 219 (100)

**MS (EI):** *m/z* (rel. int. in %) = 467 [M^+^**^•^**] (2), 250 (10), 248 (10), 217 (100), 172 (15), 168 (15), 110 (15), 91 (90)

**HR-MS (EI):** *m/z* = 465.1751 (calculated for C_26_H_32_BrN_3_: 465.1780)

**HPLC purity**: > 99 %

**Ring A homologue:**

**(±)-*N^1^*-(1-Benzylpiperidin-4-yl)-2-bromo-5,6,7,8,9,10-hexahydrocyclohepta[b]indol-6-amine (gea_139)**

C_25_H_30_BrN_3_; M*_r_* = 452.43 g/mol

306 mg (1.10 mmol) 2-bromo-7,8,9,10-tetrahydrocyclohepta[b]indol-6(5*H*)-one [^7^](#_ENREF_7), 700 mg (3.30 mmol) sodium triacetoxyborohydride, and 550 µL (2.70 mmol) 4-amino-1-benzylpiperidine are dissolved in 15 mL 1,2-dichloroethane, and 0.16 mL glacial acetic acid are added. After stirring at room temp. for 72 h 20 mL of a saturated sodium bicarbonate solution and 20 mL water are added, and the mixture is extracted with ethyl acetate (3 x 15 mL). The combined organic layers are dried over sodium sulfate, concentrated, and the residue subjected to FCC (dichloromethane/ethyl acetate/EDMA 9:1:1).

**Yield:** 195 mg (39 %), red wax

**^1^H-NMR (400 MHz, CD_2_Cl_2_):** δ (ppm) = 9.08 (br s, 1H, 5-H), 7.56 (d, *J* = 1.8 Hz, 1H, 1-H), 7.33 – 7.28 (m, 4H, 2´´´-H, 3´´´-H, 5´´´-H, 6´´´-H), 7.27 – 7.20 (m, 1H, 4´´´-H), 7.19 (d, *J* = 8.5 Hz, 1H, 4-H), 7.12 (dd, *J* = 8.5 Hz, 1.9 Hz, 1H, 3-H), 3.90 – 3.83 (m, 1H, 6-H), 3.48 (s, 2H, 1´´-H), 2.97 – 2.89 (m, 1H, 10-H), 2.84 (t, *J* = 10.3 Hz, 2H, 2´-H, 6´-H), 2.73 – 2.63 (m, 1H, 4´-H), 2.59 – 2.53 (m, 1H, 10-H), 2.18 – 2.10 (m, 2H, 7-H, 8-H), 2.09 – 2.00 (m, 2H, 2´-H, 6´-H), 1.99 – 1.91 (m, 1H, 9-H), 1.89 – 1.79 (m, 2H, 3´-H, 5´-H), 1.70 – 1.60 (m, 1H, 8-H), 1.57 – 1.42 (m, 4H, 7-H, 9-H, 3´-H, 5´-H).

**^13^C-NMR (100 MHz, CD_2_Cl_2_):** δ (ppm) = 142.6 (C-5a), 139.5 (C-1´´´), 132.2 (C-4a), 131.8 (C-10b), 129.4 (C-2´´´, C-6´´´), 128.5 (C-3´´´, C-5´´´), 127.2 (C-4´´´), 123.2 (C-3), 120.7 (C-1), 112.4 (C-4), 112.1 (C-2), 110.5 (C-10a), 63.3 (C-1´´), 55.0 (C-6), 53.9 (C-4´), 52.8 (C-2´ or C-6´), 52.6 (C-2´ or C-6´), 36.1 (C-7), 34.2 (C-3´ or C-5´), 33.5 (C-3´ or C-5´), 29.2 (C-8), 28.7 (C-9), 25.0 (C-10).

**IR (CHCl_3_ film):** $\tilde{\nu}$ (cm^-1^) = 3429, 3017, 2924, 2849, 1467, 1311, 1215, 1104, 757, 699, 668

**MS (CI):** *m/z* (rel. int. in %) = 454 [M^+^ + H] (45), 452 (45), 264 (65), 262 (80), 221 (20), 191 (100), 175 (60), 165 (20), 101 (20)

**MS (EI):** *m/z* (rel. int. in %) = 453 (2) [M^+^**^•^**], 451 (2), 279 (15), 277 (15), 262 (20), 189 (25), 175 (70), 146 (40), 91 (100), 57 (20)

**HR-MS (EI):** *m/z* = 451.1632 (calculated for C_25_H_30_BrN_3_: 451.1623)

**HPLC purity:** > 99 %

**Other synthesized compounds**

**1-(Pyrimidin-4-yl)-9*H*-carbazole (gea_82)**

C_16_H_11_N_3_; M*_r_* = 245.28 g/mol

A solution of 300 mg (1.22 mmol) 1-bromo-9*H*-carbazole [^2^](#_ENREF_2) in anhydrous THF under nitrogen is cooled to –78 °C, and treated with 2.57 mL (4.88 mmol) *tert*-butyllithium solution (1.9 M in pentane) with stirring. After coming to –20 °C over 2 h, 116 µL (1.46 mmol) pyrimidine are added, and the mixture is allowed to reach room temperature over 2 h. 30 mL water are added, followed by extraction with ethyl acetate (3 x 30 mL). The combined organic layers are dried over sodium sulfate, concentrated, and the residue purified by FCC (dichloromethane/ethyl acetate 1:1).

**Yield:** 120 mg (40 %), yellow solid

**Melting range:** 116 – 118 °C

**^1^H-NMR (400 MHz, CD_2_Cl_2_):** δ (ppm) = 11.37 (br s, 1H, NH), 9.33 (s, 1H, 2´-H), 8.75 (d, *J* = 5.6 Hz, 1H, 6´-H), 8.23 (d, *J* = 7.6 Hz, 1H, 4-H), 8.12 (d, *J* = 7.8 Hz, 1H, 5-H), 8.02 (d, *J* = 7.8 Hz, 1H, 2-H), 7.92 (d, *J* = 5.6 Hz, 1H, 5´-H), 7.60 (d, *J* = 8.1 Hz, 1H, 8-H), 7.48 (t, *J* = 7.7 Hz, 1H, 7-H), 7.31 (t, *J* = 7.7 Hz, 1H, 3-H), 7.27 (t, *J* = 7.9 Hz, 1H, 6-H).

**^13^C-NMR (100 MHz, CD_2_Cl_2_):** δ (ppm) = 164.4 (C-4´), 158.7 (C-2´), 157.4 (C-6´), 140.1 (C-8a), 139.2 (C-9a), 126.8 (C-7), 125.4 (C-4a), 124.3 (C-2), 124.1 (C-4), 122.6 (C-4b), 120.7 (C-5), 120.6 (C-6), 119.2 (C-3), 117.6 (C-1), 116.5 (C-5´), 111.6 (C-8).

**IR (KBr):** $\tilde{\nu}$ (cm^-1^) = 3372, 3049, 1596, 1577, 1531, 1498, 1466, 1453, 1423, 1391, 1344, 1324, 1262, 1225, 1183, 1125, 1010, 989, 839, 803, 744, 713, 686, 645, 622, 595, 554

**MS (CI):** *m/z* (rel. int. in %) = 246 [M^+^ + H] (100)

**MS (EI):** *m/z* (rel. int. in %) = 245 [M^+^**^•^**] (100), 191 (10), 168 (10), 157 (10), 123 (15), 109 (10), 85 (15), 64 (10)

**HR-MS (EI):** *m/z* = 245.0959 (calculated for C_16_H_11_N_3_: 245.0953)

**HPLC purity:** 86 %

**5-Iodo-4*H*-pyrido[3,2,1-*jk*]carbazol-4-one (gea_95)**

C_15_H_8_INO; M*_r_* = 345.13 g/mol

A solution of 880 mg (4.02 mmol) 4*H*-pyrido[3,2,1-*jk*]carbazol-4-one[^11^](#_ENREF_11) and 3.42 g (11.1 mmol) *N*-iodosaccharin in 20 mL DMF is stirred at 60 °C for 6 h. After cooling, 30 mL water are added, and the mixture is extracted with ethyl acetate (3 x 30 mL). The combined organic layers are dried over sodium sulfate, concentrated, and the residue purified by FCC (dichloromethane/ethyl acetate 9:1).

**Yield:** 1.33 g (96 %), yellow solid

**Melting range:** 263 – 265 °C (dec.)

^1^H-NMR (500 MHz, CDCl_3_): δ (ppm) = δ 8.87 (s, 1H, 6-H), 8.35 (dd, *J* = 7.9 Hz, 0.8 Hz, 1H, 3-H), 8.27 (dd, *J* = 7.4 Hz, 0.8 Hz, 1H, 1-H), 8.09 (dd, *J* = 7.7 Hz, 1.1 Hz, 1H, 11-H), 7.71 – 7.67 (m, 2H, 2-H, 8-H), 7.59 (td, *J* = 7.4 Hz, 1.1 Hz, 1H, 9-H), 7.48 (td, *J* = 7.6 Hz, 0.9 Hz, 1H, 10-H).

**^13^C-NMR (100 MHz, CDCl_3_):** δ (ppm) = 175.1 (C=O), 137.5 (C-7a), 137.4 (C-11c), 137.0 (C-6), 128.2 (C-9), 126.0 (C-11a), 125.8 (C-11b), 125.7 (C-2), 125.3 (C-1), 125.2 (C-3), 122.5 (C-10), 122.2 (C-11), 119.4 (C-3a), 110.4 (C-8), 88.7 (C-5).

**IR (KBr):** $\tilde{\nu}$ (cm^-1^) = 3441, 3049, 2926, 2364, 2344, 1635, 1610, 1542, 1505, 1449, 1367, 1347, 1302, 1276, 1264, 1223, 1162, 1107, 1020, 881, 871, 805, 764, 754, 718, 603, 474

**MS (CI):** *m/z* (rel. int. in %) = 346 [M^+^ + H] (15), 240 (30), 220 (10), 97 (15), 84 (40), 79 (100), 73 (20)

**MS (EI):** *m/z* (rel. int. in %) = 345 [M^+^**^•^**] (100), 219 (30), 190 (20), 163 (10), 70 (10), 54 (10)

**HR-MS (EI):** *m/z* = 344.9647 (calculated for C_15_H_8_INO: 344.9651)

**HPLC purity**: > 99 %

**Supplementary references**

1. Kent A. Complexes of polynitro-compounds. Part I. Compounds of polynitro-hydrocarbons with 1-keto-1,2,3,4-tetrahydrocarbazole. *J Chem Soc* 1935**:** 976.

2. Gehring AP, Bracher F. A convenient conversion of substituted cyclohexenones into aryl methyl ketones. *Synthesis-Stuttgart* 2012; **44**(15)**:** 2441-2447.

3. Mears AJ, Oakeshott SH, Plant SGP. The action of halogens on polycyclic indole derivatives. Part IV. Some reactions of 1-keto-1,2,3,4-tetrahydrocarbazole. *J Chem Soc* 1934**:** 272.

4. Boggs SD, Catalano JG, Gudmundsson KS, Richardson LDA, Sebahar PR. Novel cycloalkyl’b! condensed indoles. In: Patent WO 2005/023245 A1 PU, SmithKline Beecham Corp. (ed). A61K 31/404 edn2005.

5. Bahadur GA, Bailey AS, Costello G, Scott PW. Reactions of some indolyl alcohols and indolyl-olefins with arenesul-phonyl azides. *J Chem Soc, Perkin Trans 1* 1979**:** 2154.

6. Sergeev AG, Artamkina GA, Velezheva VS, Fedorova IN, Beletskaya IP. Palladium-catalyzed amination and amidation of benzo-fused bromine-containing heterocycles. *Russ J Org Chem* 2005; **41**(6)**:** 860-874.

7. Boggs SD, Gudmundsson KS, Richardson LDA, Sebahar PR. Tetrahydrocarbazole derivatives and their pharmaceutical use. In: Patent WO 2004/110999 A1 PU, SmithKline Beecham Corp. (ed). C07D 209/82 edn2004.

8. Gazengel J-M, Lancelot J-C, Rault S, Robba M. Etude des conditions d'accés aux 6,11-dihydro-5H-pyrimidino[4,5-a]carbazoles. *J Heterocycl Chem* 1990; **27**(7)**:** 1947-1951.

9. Miller CP, Bhaket P, Muthukaman N, Lyttle CR, Shomali M, Gallacher K *et al.* Synthesis of potent, substituted carbazoles as selective androgen receptor modulators (SARMs). *Bioorganic & medicinal chemistry letters* 2010; **20**(24)**:** 7516-7520.

10. Shoeb A, Anwer F, Kapil RS, Popli SP, Dua PR, Dhawan BN. N-Alkylaminocarbazoles as potential anticonvulsant and diuretic agents. *J Med Chem* 1973; **16**(4)**:** 425-427.

11. Rapoport H, Bowman DM. 4-Oxo-5, 6-dihydro-4H-pyrido carbazole and its aralkylidene derivatives. *M J Org Chem* 1959; **24**(3)**:** 324-327.
